# Low-dose metronomic cyclophosphamide complements the actions of an intratumoral C-class CpG TLR9 agonist to potentiate innate immunity and drive potent T cell-mediated anti-tumor responses

**DOI:** 10.18632/oncotarget.27322

**Published:** 2019-12-31

**Authors:** Weng In Leong, Rachel Y. Ames, Jessica M. Haverkamp, Laura Torres, Janine Kline, Ashil Bans, Lauren Rocha, Marilena Gallotta, Cristiana Guiducci, Robert L. Coffman, Mary J. Janatpour

**Affiliations:** ^1^Dynavax Technologies, Inc., Emeryville, CA 94608, USA

**Keywords:** TLR9, SD-101, cyclophosphamide, immune therapy, innate immunity

## Abstract

The synthetic oligonucleotide SD-101 is a potent and specific agonist for toll-like receptor 9. Intratumoral injection of SD-101 induces significant anti-tumor immunity in preclinical and clinical studies, especially when combined with PD-1 blockade. To build upon this strategy, we studied the enhancement of SD-101 activities by combination with low-dose cyclophosphamide, a well-characterized agent with potentially complementary activities. In multiple mouse tumor models, we demonstrate substantial anti-tumor activity of the combination, compared to each single agent. Combination therapy generated CD8+ T cell dependent immunity leading to rejection of both non-injected and injected tumors and long-term survival, even in very large tumors. Mechanistic studies encompassing global gene expression changes and characterization of immune cell infiltrates show the rapid, sequential induction of innate and adaptive responses and identify discrete contributions of SD-101 and cyclophosphamide. Importantly, these changes were prominent in tumors not injected directly with SD-101. Combination treatment resulted in creation of a permissive environment for a systemic anti-tumor immune response, including a reduction of intratumoral regulatory T cells (Tregs) and an increase in “M1” versus “M2” tumor-associated macrophage (TAM) phenotypes. Additionally, we observed increased immunogenic cell death as well as antigen processing in response to combination treatment.

## INTRODUCTION

It has long been appreciated by cancer researchers that the phenotypic heterogeneity and progressive evolution of malignant tumors minimizes the chance that any agent targeting a single molecular pathway could effectively cure advanced cancer. Indeed, not only does a given cancer cell typically usurp otherwise normal growth and anti-apoptotic mechanisms to avoid dying, it also employs mechanisms to avoid elimination by the immune system and orchestrates changes in the tumor microenvironment (TME) to ensure its survival [1].

In recent years, select subsets of patients have shown durable responses to immune checkpoint inhibitors [2–4]. Given the functional redundancies in immune checkpoints that exist, it is perhaps surprising that durable responses are observed with these single agents; but, it has been a welcome breakthrough nonetheless. However, for most tumor types, only a minority of patients respond to checkpoint blockade [2]. We have previously demonstrated in mouse tumor models that employing the innate immune system to prime a T cell response, in combination with checkpoint blockade, results in deep and durable anti-tumor efficacy [5, 6]. In cancer patients with unresectable or metastatic melanoma who have not had previous anti-PD-1 therapy, the combination of intratumoral SD-101 and systemic pembrolizumab produced a 71-78% objective response rate, higher than expected with pembrolizumab monotherapy. These high response rates were observed in both injected and non-injected tumor lesions and patients with PD-L1 negative tumors, indicating low levels of basal immune inflammation, responded as well as patients with PD-L1 positive tumors [7, 8].

SD-101 is a CpG-C class oligonucleotide that induces interferon production through engagement of toll-like receptor 9 (TLR9) in the early endosomes of plasmacytoid dendritic cells (pDCs) and induces maturation of the pDCs through engagement of TLR9 in the late endosomes of these critical antigen presenting cells [9]. Intratumorally administered SD-101 exerts its priming activity and ultimate orchestration of a systemic anti-tumor T cell response through multiple mechanisms. The production of interferon stimulates tumor cell killing by natural killer (NK) cells, with ensuing tumor antigen release, and induces chemokines that attract T cells back to the tumor bed [9–11]. The maturation of pDCs ensures optimal tumor antigen presentation to prime the anti-tumor T cell response [5]. In this way, intratumorally administered SD-101 promotes “in situ vaccination” of the tumor-burdened animal, enabling an increased response to checkpoint inhibition.

The translation of the efficacy of intratumoral SD-101 plus systemic anti-PD-1 antibody in mouse models to human subjects is encouraging; however, it is anticipated that there will be patients who fail to respond to this doublet therapy. We therefore sought to identify agents which substantially enhance the effectiveness of SD-101 that could ultimately be added to therapies based on SD-101 plus checkpoint blockade. The ideal agent would not add appreciable toxicity, but would facilitate a TME optimally permissive for an SD-101-primed immune response. To this end, we identified low-dose, metronomic (repeated low doses, rather than a single standard dose) cyclophosphamide as an ideal candidate to mediate mechanisms that would be complementary to SD-101 and potentially further enhance responses to checkpoint inhibitors, once combined in the clinic.

Low-dose metronomic cyclophosphamide (CY) has been explored for more than a decade in the clinic – first for its anti-angiogenic properties [12–14] and later for its increasingly appreciated ability to decrease T regulatory (Treg) cells [15, 16]. Additional impacted biological activities have been described, such as increased interferon production, induction of immunogenic cell death, increases in effector T cells, and increases in functional NK cells [15, 17–19], likely to be complementary to SD-101 activity by virtue of modulation of the TME. One described activity of low-dose cyclophosphamide that would possibly impede an optimal immune response is the induction of myeloid-derived suppressor cells (MDSCs) [20, 21]. It should be noted, however, that CY-induced changes in the myeloid compartment are complex; for example, they also include induction of dendritic cells, and may depend on CY dose [22, 23].

Here, we demonstrate that non-leukodepleting low-dose cyclophosphamide combined with SD-101 confers durable anti-tumor responses. By administering SD-101 locally (either intratumorally in sub-cutaneous tumors or by inhalation to tumor-bearing lungs), rather than systemically, we demonstrate that localized SD-101 injection combined with systemically administered low-dose cyclophosphamide confers an anti-tumor response at non-injected sites. Furthermore, both early and sustained changes in the TME observed in the combination treatment mechanistically align with the observed durable systemic anti-tumor immune response. Lastly, we show that the efficacy of low-dose CY and SD-101 combination treatment is ultimately dependent on CD8+ T cells.

## RESULTS

### Low dose, metronomic CY decreases tregs, increases activated NK cells and modulates the myeloid population in tumor-burdened mice

**Figure 1:**
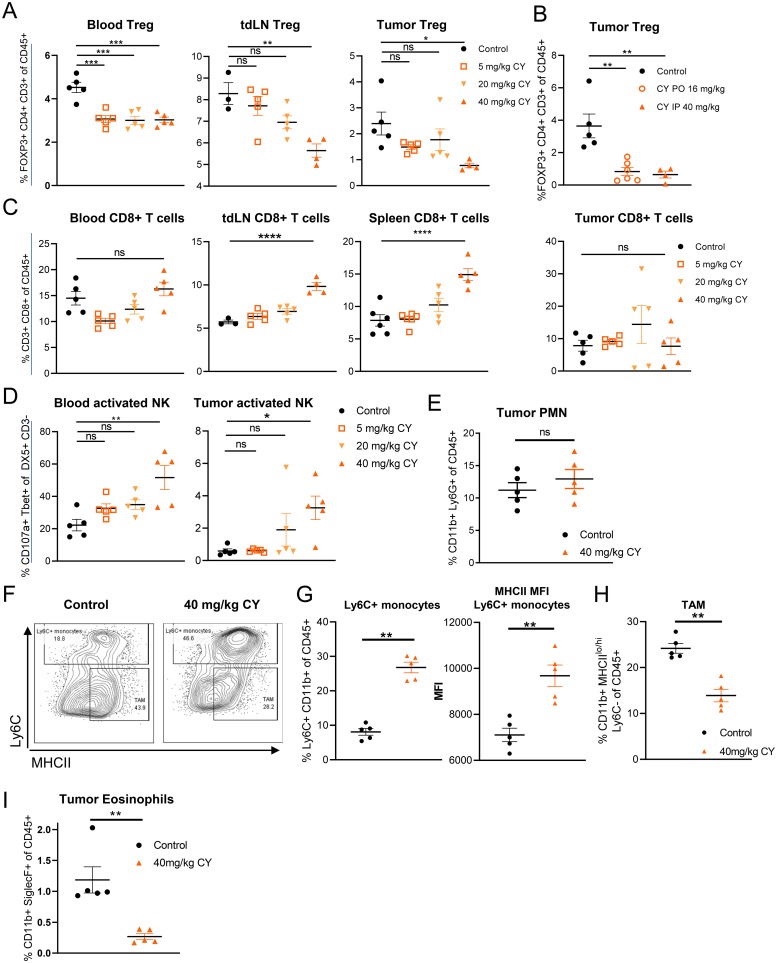
Low dose, metronomic cyclophosphamide decreases Tregs, increases activated NK cells and modulates the myeloid population in tumor-burdened mice. Data reflect two weeks of CY treatment, twice weekly (i.p.) or 5 doses weekly (p.o.) dosing in the syngeneic CT26 tumor model. **(A)** Frequency of Tregs in response to increasing doses of CY (i.p.). **(B)** Frequency of intratumoral Tregs following either p.o. or i.p. CY dosing. **(C)** Frequency of CD8+ T cells in response to increasing doses of CY (i.p.). **(D)** NK cell activity in response to increasing doses of CY (i.p.). **(E)** Frequency of intratumoral PMN following 40 mg/kg CY (i.p.) treatment. **(F)** Gating scheme for Ly6C+ CD11b+ myeloid cells and TAMs. **(G)** Frequency and MHCII expression of Ly6C+ CD11b+ myeloid cells in the tumor in response to 40 mg/kg CY. **(H)** Frequency of intratumoral MHCII^lo-hi^ Ly6C- CD11b+ TAMs in response to 40 mg/kg CY. **(I)** Frequency of eosinophils following 40 mg/kg CY (i.p.) treatment. Data show the mean ± SEM. ^*^ indicates P ≤ 0.05, ^**^ P ≤ 0.01, ^***^ P ≤ 0.001, and ^****^ P ≤ 0.0001. Representative graphs shown of one (A–D, I), or at least two (E–H) independent experiments.

Although cyclophosphamide has been previously shown to have multiple effects on the immune system [15], it was important to identify the minimum dose of CY that would allow us to observe its effects on immune cells without depletion of CD8+ T cells, which are central to the anti-tumor activities of SD-101 [5]. We determined that either 40 mg/kg CY, given i.p. twice per week for two weeks, or 16 mg/kg CY given p.o. five days per week for two weeks reduced the frequency of Tregs to a similar extent both systemically and in CT26 subcutaneous tumors (Figure 1A, 1B). Importantly, we did not observe depletion of CD8+ T cells in tumor or blood and observed an increase in frequency of CD8+ T cells in some central lymphoid organs such as the spleen and lymph nodes, confirming prior reports (Figure 1C) [16, 24– 26].

In response to low-dose CY, we observed a reduction in the frequency of total NK cells in the tumor, relative to saline control treated tumor-burdened mice (Supplementary Figure 1); however, a higher proportion of these NK cells were activated in low-dose CY-treated tumor and blood, as indicated by their expression of TBET and CD107a, reaching a statistical significance at a dose of 40 mg/kg (Figure 1D). Increased NK cell function has also been observed in patients treated with low-dose metronomic cyclophosphamide [15]. Dosing regimens totaling less than 80 mg/kg per week were not sufficiently robust in their modulation of Tregs and activated NK cells (Figure 1A–1D), so 40 mg/kg twice per week i.p. or 16 mg/kg five times per week p.o. were the low-dose CY regimens used for all subsequent experiments.

We observed several changes in the myeloid compartment within tumors in response to low-dose CY. Cyclophosphamide has been shown to increase both MDSC subtypes, polymorphonuclear MDSCs (PMN-MDSCs) as well as monocytic MDSCs (Mo-MDSC) [27–29]. We did not observe an increase in Ly6G+ PMN-MDSCs in the tumor microenvironment (Figure 1E). In contrast, Ly6C+ CD11b+ monocytes did increase in frequency in tumors (Figure 1G). The expression of MHCII on these cells was also elevated, consistent with further differentiation towards immature dendritic cell (DC) or macrophage phenotypes (Figure 1G). However, despite this increase in potential precursors, we saw a reduction in tumor-associated macrophages (TAMs; Ly6C-CD11b+ MHCII^lo/hi^) in response to low-dose CY (Figure 1H). Interestingly, tumor eosinophils, which have been shown to promote M2 macrophage development by secretion of IL-13 [30], were significantly depleted with low-dose CY treatment (Figure 1I). Therefore, low-dose CY induces changes in the myeloid compartment that reduce some immunosuppressive cell types (TAMs, eosinophils), while increasing MHCII+ monocytes (Figure 1G–1I).

### Intratumoral SD-101 combined with low-dose CY effectively inhibits growth of both injected and non-injected tumor sites and promotes survival

**Figure 2:**
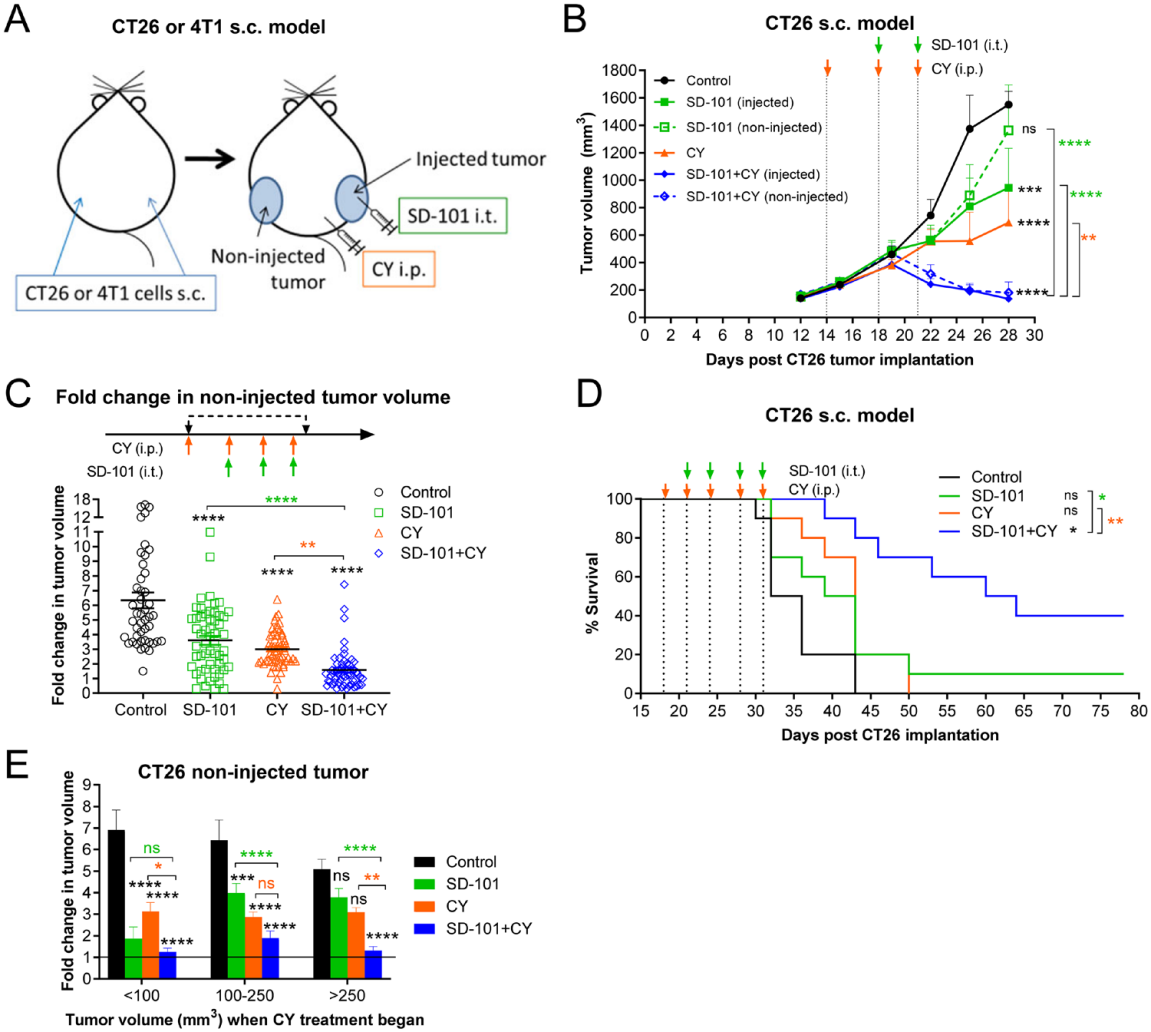
Intratumoral SD-101 combined with low-dose CY effectively inhibits growth of both injected and non-injected tumor sites and promotes survival. **(A)** Illustration of the syngeneic mouse tumor models. Briefly, CY (40 mg/kg) was given i.p. and SD-101 (50 μg/injection) was given i.t., twice weekly. **(B)** Tumor growth at the injected and non-injected sites was monitored (n= 24/group at d12 and d15, n=18/group at d19, n=12/group at d22 and n=6/group at d25 and d28; mice were removed for mechanistic evaluation during the course of the study). **(C)** Cumulative data of the fold increase in non-injected tumor volume following 4 doses of CY and 3 doses of SD-101 from 7 experiments, n=45-57/group. **(D)** Long-term survival of mice bearing CT26 s.c. tumor on both flanks, receiving saline, monotherapy (CY i.p. or SD-101 i.t.) or combination therapy as illustrated in (A). Experiment schedule was similar as in (B) but with longer treatment times, as indicated on the survival curve (D), n=10/group. **(E)** Data from (C) was divided according to the tumor size at the start of CY treatment (<100 mm^3^, 100-200 mm^3^ and > 250 mm^3^). Resulting groups ranged from 9 to 27 mice, with an average of 18 mice per group. Data show the mean ± SEM, ^*^ compared with control, ^*^ compared with SD-101, ^*^ compared with CY. ^*^ indicates P ≤ 0.05, ^**^ P ≤ 0.01, ^***^ P ≤ 0.001, and ^****^ P ≤ 0.0001.

To determine the antitumor efficacy of the combination of intratumoral SD-101 plus systemic low-dose CY, we utilized the syngeneic mouse CT26 and 4T1 tumor models in BALB/c mice. In these experiments, subcutaneous tumors were implanted at two sites, but only one site was injected with SD-101 (Figure 2A). This design allowed independent assessment of local versus systemic effects of the intratumoral TLR9 agonist treatment. Tumor-bearing mice were divided into four treatment groups: (1) control, (2) SD-101, (3) low-dose 40 mg/kg CY and (4) combination treatment with SD-101 and low-dose CY. Low-dose CY (40 mg/kg body weight) was given i.p. twice weekly and treatment began when the average tumor volume was ~200 mm^3^ (~day 14-19). SD-101 (50 μg/injection) was subsequently injected intratumorally into the right flank tumors (injected tumor) twice weekly beginning ~3 days later, when average tumor volume reached ~300-400 mm^3^. Tumor size in both SD-101 injected tumors and non-injected tumors were monitored for response to treatment.

In preliminary experiments, we found that starting CY treatment first was more effective than initiating both treatments simultaneously (data not shown). In the CT26 model, combination treatment with as few as three low-doses of CY and two doses of SD-101 was sufficient to induce inhibition of tumor growth as compared to control at both injected and non-injected sites (Figure 2B). In Figure 2C, fold-change in growth relative to each starting tumor size was calculated after four doses of CY and/or three doses of SD-101. This measurement showed the fold-change in non-injected tumor volume averaged 6, 3.6, 3 and 1.6-fold in the control, SD-101, CY and combination groups, respectively (Figure 2C). Thus, the combination treatment resulted in significantly greater inhibition of CT26 tumor growth than the respective monotherapy treatments, often causing complete regression of established tumors. Also in this model, the combination therapy administered for two and half weeks substantially promoted long-term survival (Figure 2D).

Because implanted tumors do not grow uniformly across mice, it was possible to ask whether the tumor size at the start of treatment was an important variable in observing the degree of increased efficacy conferred with the combination treatment. Response data from seven experiments was divided according to the tumor size at the start of CY treatment on day 14-19, the time at which CY treatment was initiated in groups being treated with CY. In smaller tumors (<100 mm^3^ when treatment began), both monotherapies were quite effective, with the combination showing only a small improvement in response. However, with increasing tumor sizes, the monotherapies became increasingly less effective, and the combination showed increasing benefit over either single agent. This was particularly clear with the largest tumors (>250 mm^3^), in which neither monotherapy was significantly different than the control group, while the combination therapy very effectively inhibited tumor growth (Figure 2E).

### Localized SD-101 combined with low-dose CY effectively inhibits tumor growth in multiple mouse tumor models

**Figure 3:**
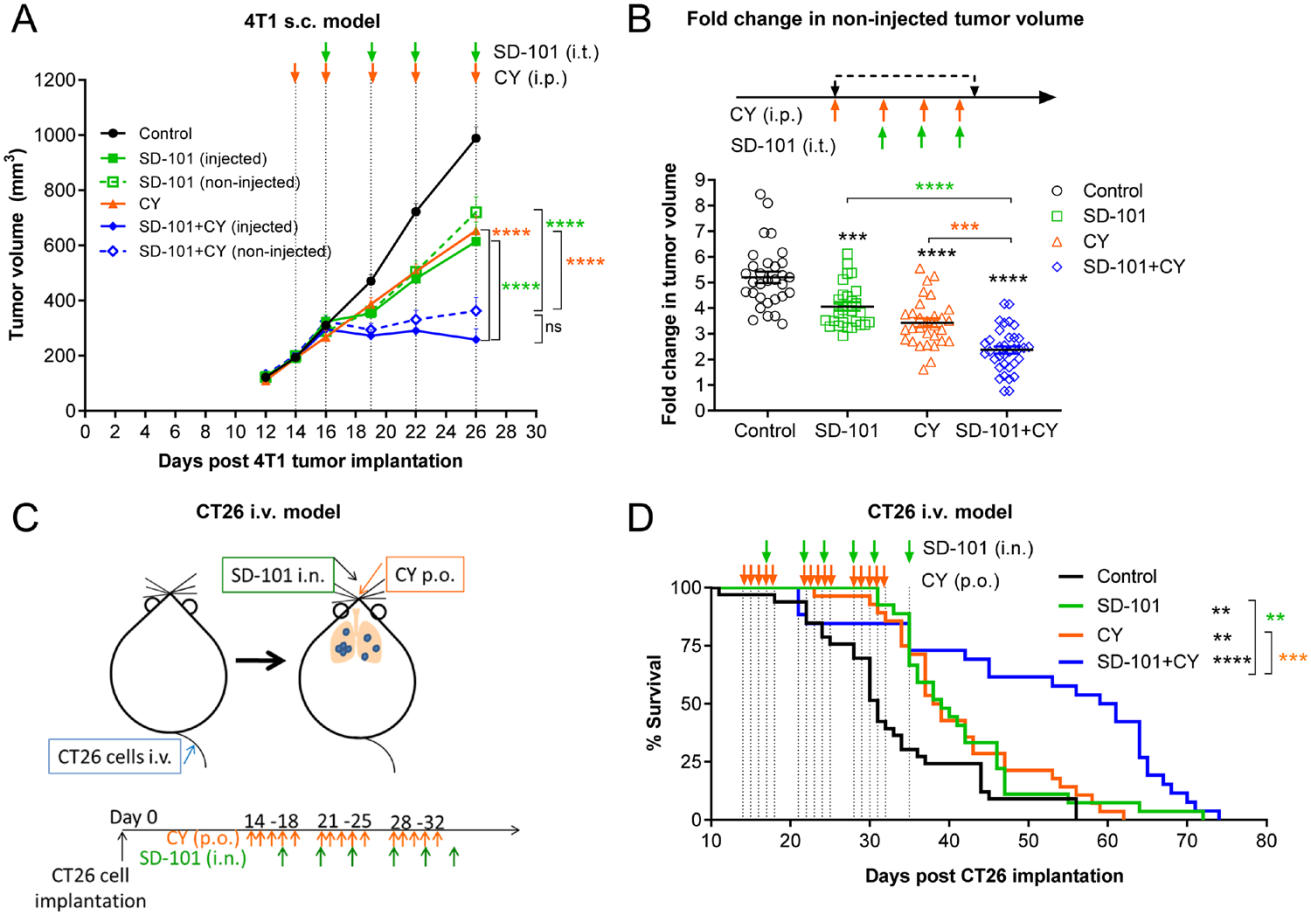
Localized SD-101 combined with low-dose CY effectively inhibits tumor growth in multiple mouse tumor models. **(A)** Tumor growth at the injected and non-injected flanks of 4T1 s.c. tumor-bearing mice, n=10/group. Illustration of the syngeneic mouse 4T1 tumor model is found in Figure 2A and treatment schedule is illustrated in (A). **(B)** Cumulative data of fold increase in 4T1 non-injected tumor volume following 4 doses of CY and 3 doses of SD-101 from 3 experiments, n= 27-35/group. **(C)** Illustration of CT26 i.v. tumor-burdened lung model. CT26 cells were injected i.v. to allow dissemination into the lungs. Mice received SD-101 i.n. (10 μg/50 μl saline) biweekly, and/or CY p.o. (16 mg/kg body weight) treatment 5 times per week. **(D)** Mice bearing CT26 lung tumors from (C), n=26-33/group were monitored for long-term survival. Data shown represents the combination of 3 independent experiments, n=34-36/group. Data show the mean ± SEM, ^*^ compared with control, ^*^ compared with SD-101, ^*^ compared with CY. ^*^ indicates P ≤ 0.05, ^**^ P ≤ 0.01, ^***^ P ≤ 0.001, and ^****^ P ≤ 0.0001.

To ensure the effects of the combination therapy were not cell line specific, a distinct model was also evaluated, using the schema described in Figure 2A. In the highly immunosuppressive sub-cutaneous 4T1 mouse tumor model, the combination treatment also significantly suppressed tumor growth at both SD-101 injected and non-injected sites (Figure 3A). Cumulative data from three experiments showed significantly lower fold change in non-injected tumor volume post treatment with combination therapy compared to the monotherapies (Figure 3B).

To ensure the efficacy would translate to tumor-bearing organs, models of tumor-burdened lung were evaluated. Mice were injected intravenously (i.v.) with CT26 cells via tail vein (shown in Figure 3C), resulting in extensive tumor seeding in lung. SD-101 (10 μg) was delivered to the lung intranasally (i.n.) as previously described [6], twice weekly, and low-dose CY (16 mg/kg body weight) was given orally, five doses weekly. Combination treatment significantly improved the survival of mice bearing CT26 tumors in the lung compared with mice receiving monotherapy treatments (Figure 3D). A similar result was observed in a 4T1 lung metastasis model [6], in which the combination of systemic low-dose CY plus inhaled SD-101 conferred increased survival in 4T1 tumor-bearing mice (Supplementary Figure 2A, 2B).

Collectively, the combination of systemic low-dose CY and intratumoral SD-101 elicited strong local and systemic anti-tumor responses leading to significant tumor inhibition and increased survival in both CT26 and 4T1 syngeneic mouse tumor models.

### Gene expression changes to the TME demonstrate sequential development of innate and adaptive immune responses

**Figure 4:**
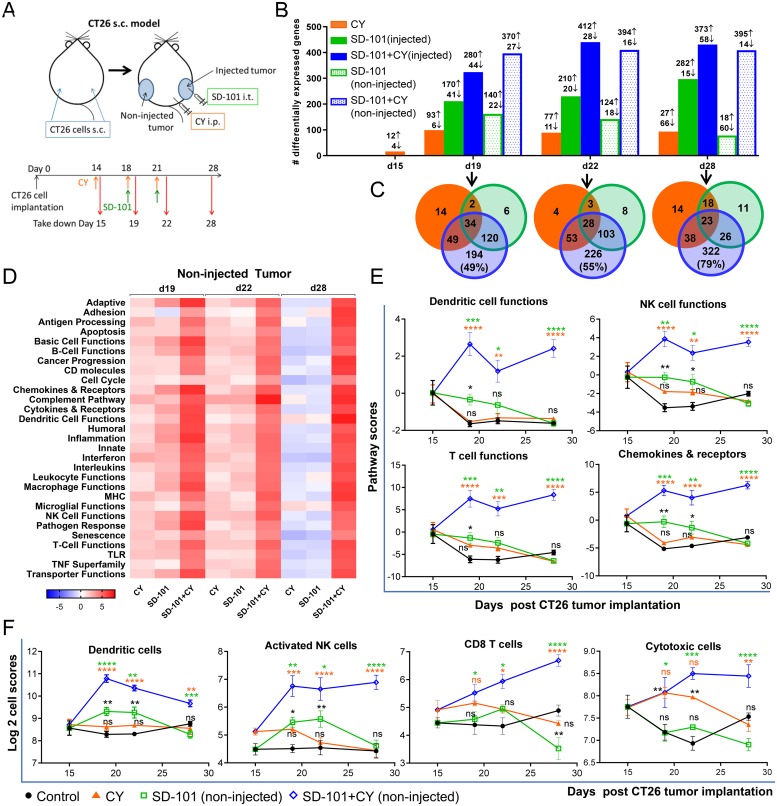
Gene expression changes to the TME demonstrate sequential development of innate and adaptive immune responses. **(A)** CT26 cells were implanted s.c. in both flanks (n=4-5/group). CY (40 mg/kg) was given i.p. and SD-101 (50 μg/injection) was injected into the right flank (injected site). RNA was extracted from tumors as indicated in the experimental schema (A). **(B)** Number of differentially expressed (DE) genes in the treatment groups that exhibit a log_2_ fold change >0.6 (>1.5 fold change) and P<0.05 compared to control group at the indicated time points. **(C)** Venn diagrams of the number of overlapping DE genes of the non-injected tumors between treatment conditions. **(D)** Heatmap of directed global significance scores (non-injected tumor), which display the extent to which a gene sets’ genes are up or down-regulated with the treatments relative to control at that time point. **(E)** Pathway scores (non-injected tumor), which summarize the data from a pathway’s genes with a single score, of select functional pathways. **(F)** Cell type scores (non-injected tumor), which measure the intratumoral abundance of immune cells using specific gene signatures. Data are mean ± SEM, ^*^ compared with control, ^*^ compared with SD-101, ^*^ compared with CY. ^*^ indicates P ≤ 0.05, ^**^ P ≤ 0.01, ^***^ P ≤ 0.001, and ^****^ P ≤ 0.0001.

Given the clear increase in efficacy of the combination of systemic low-dose CY and intratumoral SD-101 relative to either monotherapy, we sought to understand the factors contributing to the functional complementarity by examining the global gene expression changes in the tumors. Mice bearing CT26 s.c. tumors on both flanks were divided into four groups (1) control, (2) SD-101 (3) CY and (4) combination. Low-dose CY (40 mg/kg) was given i.p. at days 14, 18, and 21, and SD-101 was injected into the right flank (injected tumor) at days 18 and 21 post-CT26 cell implantation (Figure 4A). To understand the kinetics of the gene expression changes in the tumor induced by the respective treatments, tumors were collected and RNA was extracted during treatment at days 15, 19 or 22 or one week after the last treatment at day 28. Gene expression profiling of the tumors was performed on mRNA from injected and non-injected tumors using the PanCancer Immune profiling panel from NanoString™. Multiple types of gene expression analyses were performed on these data.

Significant gene expression changes were observed in the injected and non-injected tumors of the combination group after just two low-doses of CY and one dose of SD-101 treatment (d19; Figure 4B). To assess systemic antitumor immunity generated by the combination, we focused on the global changes in the non-injected tumor sites. At day 19, the number of genes differentially expressed (DE) relative to untreated controls in the CY, SD-101 and the combination group were 99, 162 and 397, respectively (non-injected tumor) compared to control (Figure 4B), with a large proportion of genes differentially expressed only in the combination. The Venn diagrams show the number of overlapping DE genes in the non-injected tumors from all treatment groups relative to control (Figure 4C). A substantial number of genes were differentially expressed only in the combination, with 49% (d19), 55% (d22) and 79% (d28) of the DE genes in the combination unique to the combination treatment (Figure 4C, Supplementary Table 1). Comparing monotherapies, SD-101 alone induced more gene changes than low-dose CY alone. The monotherapies produced distinct patterns of gene expression changes; only 22% of the DE genes in SD-101 were in common with low-dose CY monotherapy at both day 19 and day 22.

Directed global significance scores showed robust up-regulation of numerous immune-related signatures, including enhanced antigen processing, DC, NK, B, and T cell functions with the combination treatment in non-injected tumors (Figure 4D). Similar results were observed in injected tumors, although changes in the SD-101 only group were more prominent (Supplementary Figure 3A). The enhanced antitumor immunity mediated by low-dose CY began soon after the first SD-101 treatment (d19). Interestingly, synergistic gene expression changes with combination treatment were observed in the non-injected tumor one week after stopping treatment, a time point at which the respective monotherapies showed minimal gene expression changes (d28; Figure 4D). This observation is consistent with the durability of anti-tumor responses observed with combination therapy (e.g. Figure 2D).

To better understand the systemic effects of the combination treatment on immune cell functions (pathway scores), non-injected tumors were evaluated for effects of the combination compared to the respective monotherapies. SD-101 treatment alone, but not low-dose CY alone, led to up-regulation of DC, NK, B and T cell functions as well as chemokines and their receptors at day 19, as indicated by the pathway scores, diminishing at d22 and 28 (Figure 4E & Supplementary Figure 4A). Combination treatment, however, not only further up-regulated these gene sets, but resulted in maintained elevation even one week after the last treatment (d28) (Figure 4E). Other pathway scores significantly upregulated by the combination treatment in the non-injected tumors included antigen processing, MHC, complement pathway, TNF superfamily and interferon (Supplementary Figure 4A).

Consistent with the increased DC, NK and T cell functions, Nanostring™ analysis revealed large and significant increases in non-injected tumor infiltrating immune cell marker genes (cell type scores) for DCs, activated NK cells, T cells, CD8+ T cells, and cytotoxic cells resulting from combination therapy compared to the monotherapies, while no change occurred in B cell signature scores (Figure 4F & Supplementary Figure 4B). Combination treatment increased DC and activated NK cell gene signatures as early as d19, whereas T cell, CD8+ T cell and cytotoxic cells were increased at the later time points (d22 and 28), a pattern consistent with the proposed mechanism of action of SD-101 to directly activate an innate immune response leading to a subsequent increase in adaptive immunity. Cell type scores suggested that SD-101 alone had a greater impact on DC and activated NK cell tumor infiltration and that low-dose CY drove the cytotoxic cell marker genes early in the course of treatment. Heatmaps showing relative expression levels of individual genes comprising the DC-, NK- and T cell- functional gene signatures, as well as the cytotoxic gene signature in the non-injected tumors are found in Supplementary Figure 5.

Expression changes of selected genes implicated in anti-tumor immunity were examined using real time qPCR both to validate Nanostring™ results and to understand the temporal nature of the expression of these key factors (Supplementary Figure 6). In accord with Nanostring™ data, qPCR analysis in non-injected tumors treated with the combination demonstrated significant up-regulation of Th1 cytokines and chemokines (*Tnf, Il1b, Il6, Il18, Ifng, Ccl5, Cxcl9* and *Cxcl10*), CD8 (*Cd8b*), a key costimulatory molecule (*Cd80)* and a cell adhesion molecule known to be important for T cell recruitment to the tumor bed (*Icam1*). Conversely, down-regulation of the tumor promoting cytokine *Il11* with combination therapy was observed. The timing of maximal gene expression changes differed amongst respective genes (Supplementary Figure 6). Induction of *Tnf, Il1b, Il6, Ccl5, Cxcl10* and *Cd80* in response to the combination treatment reached their maximum early, at d19 (after one dose of SD-101 and two low-doses of CY). Induction of these genes was likely driven by SD-101, as they were evident in the SD-101 monotherapy treatment. In contrast, up-regulation of *Cd8b*, *Ifng* and *Il18* with combination treatment was highest at d28, coinciding with tumor regression.

**Figure 5:**
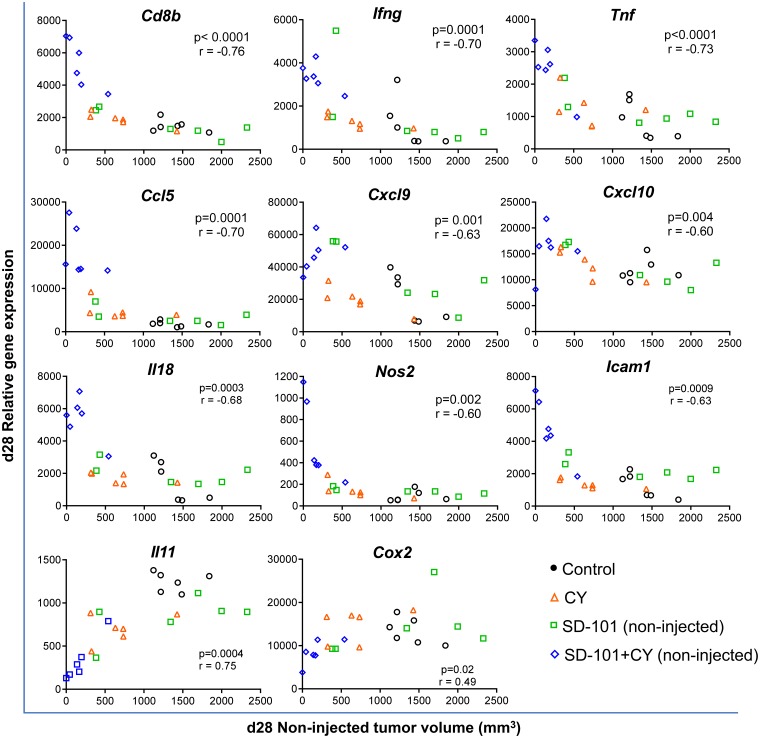
Tumor volume in response to combination treatment correlates negatively with genes of anti-tumor immunity and positively with pro-tumorigenic molecules. Tumors were harvested at d28, 7 days after the last treatment at d21 (Figure 4A). Gene expression was measured by real time qPCR. Pearson correlation test was applied to calculate the correlation between gene expression levels in the non-injected tumor and the tumor volume of the non-injected site at d28, n=6/group.

Using this qPCR gene expression data, the correlation between non-injected tumor volume and gene expression at d28 was determined. Tumor regression in response to combination treatments correlated strongly with elevated tumor expression of *CD8b, Ifng, Tnfa, Ccl5* and *Il18* in the non-injected tumors (Figure 5). Similar correlations were observed between tumor volume and the expression of Th1 chemokines (*Cxcl9, Cxcl10*), adhesion molecules (*Icam1*) and the M1 macrophage marker *Nos2*. In contrast, increased tumor growth inhibition correlated with decreased expression of pro-tumorigenic molecules *Cox2* and *Il11*. Overall, the global gene expression changes modulated by the combination treatment strongly favored systemic antitumor immunity, resulting in tumor growth inhibition at the non-injected site.

### SD-101 and low-dose CY combination increases immunogenic cell death and APC function within the tumor

**Figure 6:**
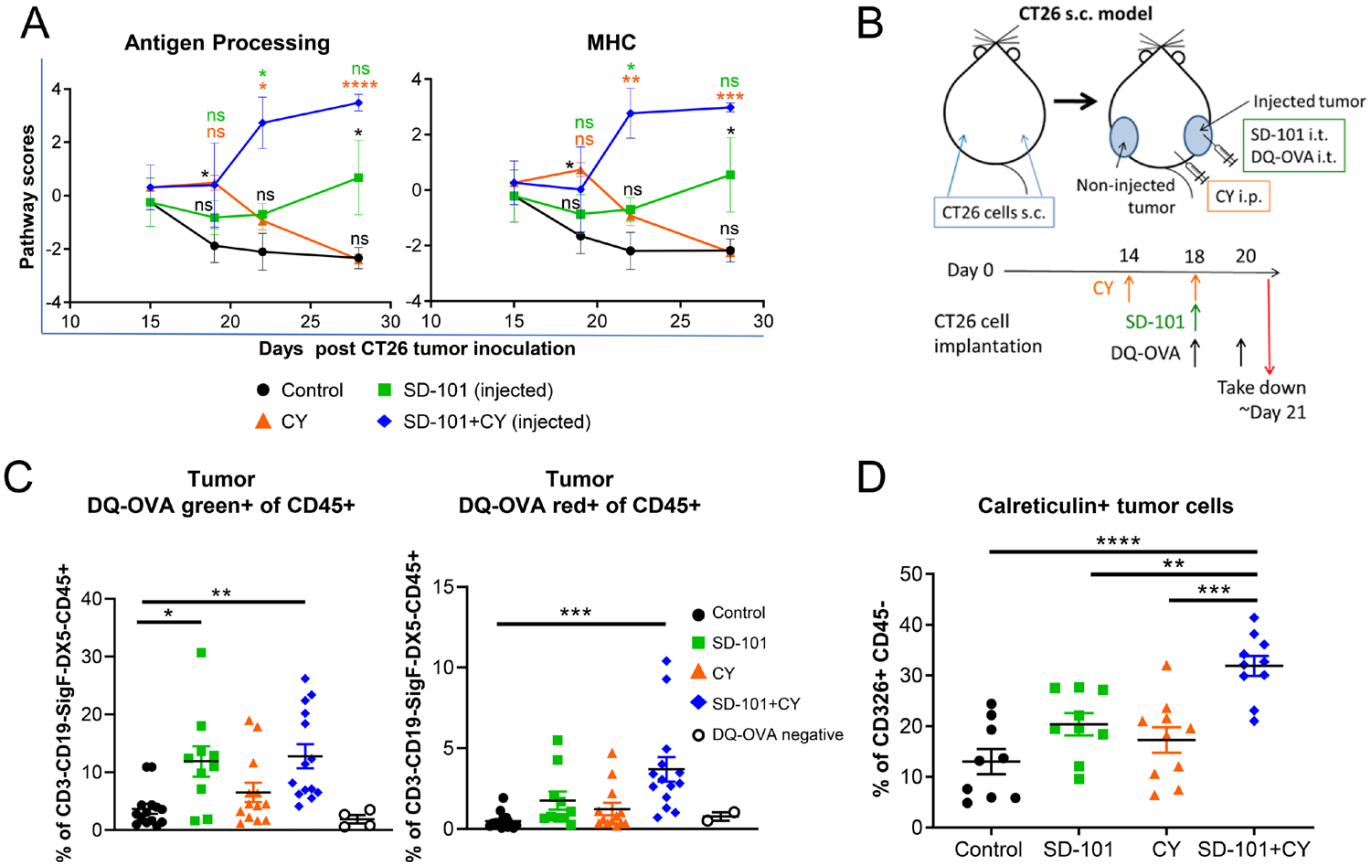
SD-101 and low-dose CY combination increases immunogenic cell death and APC function within the tumor. **(A)** Pathway scores of antigen processing and MHC functional pathways in the injected tumors. **(B)** Experimental design for DQ-OVA tracking of antigen processing *in vivo*. **(C)** Percentage of CD3-CD19-SiglecF-DX5-CD45+ immune cells positive for processed DQ-OVA (DQ-OVA green+) or for highly accumulated processed DQ-OVA (DQ-OVA red+) in injected tumors following treatment with SD-101 and/or CY as indicated. Graphs reflect two combined independent experiments, n=10-14/group. **(D)** CD326+ CD45- cells showing surface calreticulin following treatment with SD-101 and/or CY as indicated. Data are mean ± SEM, n=9-10/group. ^*^ indicates P ≤ 0.05, ^**^ P ≤ 0.01, ^***^ P ≤ 0.001, and ^****^ P ≤ 0.0001. Representative graph of two independent experiments.

To identify the early, primary mechanisms by which low-dose CY complements SD-101 to elicit the strong antitumor efficacy, the gene sets with the highest global significance scores exhibited by low-dose CY alone at day 19 were examined. These included antigen processing, MHC, complement, and interferon signatures (Figure 4D and Supplementary Figure 3B–3E). Low-dose CY had a stronger impact on the antigen processing and MHC pathway scores compared to SD-101 at d19 in the injected tumor where T cell priming originates (Figure 6A). After two doses of CY at day 19, CY monotherapy increased expression of genes involved in MHCI (*H2-K1, H2-Q2, H2-T23*, *Psmb8*, *Psmb9*, *Tap1, Tap2*, *Tapbp*) and MHCII (*H2-Dma, H2-Eb1*, *Cd74*) pathways. As a result, combination treatment induced up-regulation of more genes in the antigen processing and the MHC gene sets than the respective monotherapies alone (Supplementary Figure 3B and 3C), and this also corresponded to significantly increased pathway scores for antigen processing and MHC in response to combination therapy at d22 and d28 (Figure 6A).

We hypothesized that the combination of these agents might thereby optimally increase antigen uptake and processing in tumors *in vivo*. After a lead-in dose of CY, we injected DQ-OVA intratumorally alongside a single combination treatment with SD-101 and low-dose CY (Figure 6B). DQ-OVA is a fluorogenic substrate that is self-quenched when intact, unveils green fluorescence when proteolytically cleaved, and emits red fluorescence when it accumulates in organelles at a high concentration. We observed increased proteolytic cleavage of DQ-OVA by CD45+ cells in SD-101 and combination treated tumors, and increased accumulation of proteolytically cleaved DQ-OVA in combination-treated tumors (Figure 6C), consistent with increased antigen processing in the tumor.

Cyclophosphamide is known to stimulate immunogenic cell death, a process that contributes to activation, maturation, and trafficking of antigen presenting cells [31]. Using calreticulin exposed on the surface of tumor cells, which operates as an “eat me” signal to stimulate uptake of tumor antigens by APCs, we showed that the combination treatment increased this hallmark of immunogenic cell death (Figure 6D). Taken together, these data suggest that combination of SD-101 and low-dose CY treatment results in increased immunogenicity and antigen processing in the tumor microenvironment.

### Combination therapy activates monocytes and shifts toward M1 macrophage development

**Figure 7:**
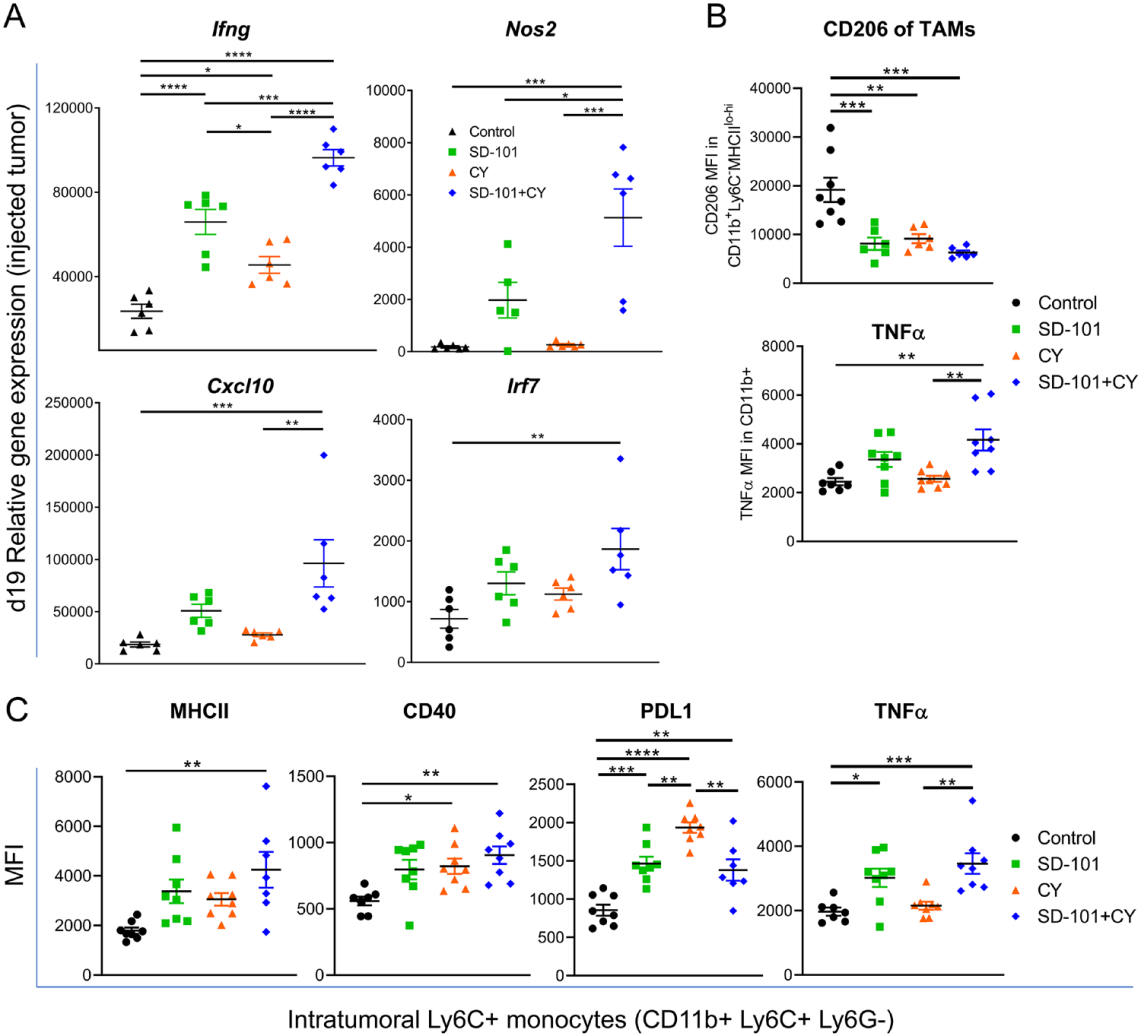
Combination therapy activates monocytes and shifts toward M1 macrophage development. **(A)** Relative expression, measured by qPCR, of M1-associated genes in the injected tumor at d19 (from Figure 4A) in response to indicated treatments. Representative graphs of two independent experiments, except for *Nos2* (one experiment), n=5-6/group. **(B)** CD206 (MFI) in CD11b+ MHCII^lo-hi^ TAMs and TNFɑ (MFI) in CD11b+ cells. **(C)** Expression of MHCII, CD40, PDL1, and TNFɑ on Ly6C+ monocytes (CD11b+Ly6C+Ly6G-). (B-C) Experiments reflect tumors collected following 5 doses of i.p. CY and 4 doses of i.t. SD-101, given twice weekly. Data are mean ± SEM, n=8/group. ^*^ indicates P ≤ 0.05, ^**^ P ≤ 0.01, ^***^ P ≤ 0.001, and ^****^ P ≤ 0.0001. If comparison is not labelled, it is not statistically significant (P>0.05). Representative graphs of two independent experiments.

A major potential source of immunosuppression in the tumor are myeloid cells, including TAMs and MDSCs [32, 33]. TAMs are a heterogenous mix of cells expressing markers of both M1 and M2 macrophages [30]. However, TAMs associated with poor prognosis are thought to more closely resemble M2 macrophages, and repolarization of TAMs toward an activated, M1-like phenotype has been shown to aid in tumor eradication [34, 35]. We first measured gene expression in the injected tumor tissue to determine if any genes associated with M1 macrophages versus M2 changed in response to our combination treatment at d19. We saw a significant increase in expression of *Ifng* in response to SD-101 or low-dose CY, with significantly higher levels elicited by combination low-dose CY and SD-101 treatment versus either single agent (Figure 7A). M1 macrophages also express higher levels of *Nos2*, *Cxcl10,* and *Irf7* and expression of each of these genes increased in the injected tumor in response to combination treatment (Figure 7A). To determine the overall balance of M1- to M2-associated genes, fifteen M1-related genes and seven M2-related genes, previously identified as markers for these phenotypes and which are found within the Nanostring™ gene set [36] were evaluated, and the ratio of the geometric mean of M1 to M2 genes was calculated. We observed a significantly higher M1/M2 gene expression ratio with the combination treatment compared to control in both the injected and non-injected tumors (Supplementary Figure 3G). We next sought to determine whether the myeloid cells themselves were producing M1- or M2-like factors. Flow cytometric analysis showed that CD11b+ MHCII^lo-hi^ TAMs significantly downregulated the M2 marker CD206. Additionally, the M1 macrophage-associated, M2-macrophage-inhibiting cytokine TNFα was upregulated in total CD11b+ myeloid cells in response to combination treatment (Figure 7B).

We also characterized molecular changes in Ly6C+ monocytes to further understand how the combination treatment impacted the tumor-associated myeloid compartment. Ly6C+ CD11b+ monocytes upregulated CD40 and MHCII in response to combination treatment, consistent with differentiation into immature antigen-presenting cells (Figure 7C). In addition, we saw increases in TNFα, as we had seen in total CD11b+ myeloid cells, and in PD-L1, consistent with upregulation of interferon-induced pathways seen in the gene expression data (Figure 7C). These data suggest that SD-101 in combination with low-dose CY can further enhance the ability of SD-101 to activate inflammatory monocytes.

### SD-101 and low-dose CY combination drives increased activity of CD8+ T cells, which are required for efficacy of treatment

**Figure 8:**
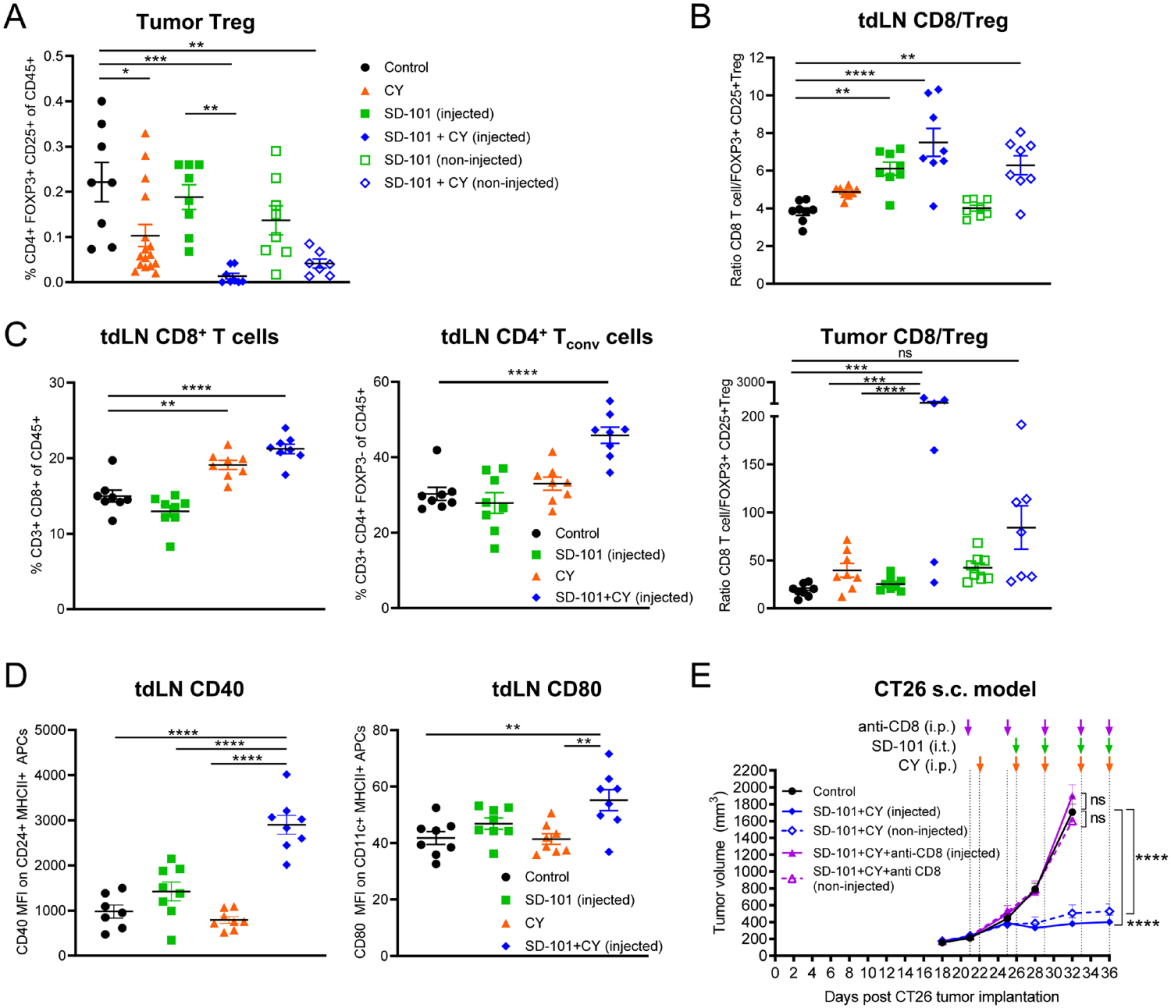
SD-101 and low-dose CY combination drives increased activity of CD8+ T cells, which are required for efficacy of treatment. **(A)** Frequency of intratumoral Tregs in response to indicated treatments, n=8-16/group. **(B)** Ratio of CD8+ T cells to Tregs in the tdLN and tumor. **(C)** Frequency of CD8+ T cells and CD4+ Tconv (CD3+ CD4+ FOXP3-) in the tdLN. (A–C) Tumors and tdLN collected following 5 doses of i.p. CY and 4 doses of i.t. SD-101, given twice weekly. **(D)** Expression of costimulatory molecules on APCs in the tdLN. CD40 (MFI) was measured on CD24+ MHCII+ cells that were CD3-CD19-SiglecF-Ly6G-. CD80 (MFI) was measured on CD11c+ MHCII+ cells that were CD3-CD19-SiglecF-Ly6G-. Tumor draining LN collected 2 days following the last dose of 2 doses of i.p. CY given twice weekly and 1 dose of i.t. SD-101 (approx. d19). **(E)** Tumor volume resulting from depletion of CD8+ T cells during combination treatment. Data are the mean ± SEM, n=10/group. ^*^ indicates P ≤ 0.05, ^**^ P ≤ 0.01, ^***^ P ≤ 0.001, and ^****^ P ≤ 0.0001. (A–E) Representative graphs of at least two independent experiments.

CY has been shown to deplete Tregs in the TME. We observed Treg depletion in response to low-dose CY compared with control tumor, and Tregs were significantly depleted in response to the combination treatment versus SD-101 alone in the injected tumor (Figure 8A). The ratio of effector T cells to Tregs is a major factor in determining the ability of T cells to respond to stimuli [37] and higher ratios in the tumor correlate with improved prognosis in a variety of tumor types [38]. We observed a significant increase in the CD8+ T cell to Treg ratio in both SD-101 injected tumors and tumor-draining lymph nodes in the context of combination treatment (Figure 8B).

In addition to depletion of Tregs, we saw changes consistent with increased priming in tumor-draining lymph nodes. The frequency of total CD4+ Tconv cells and CD8+ T cells in tumor-draining lymph nodes was increased by combination treatment. This was accompanied by an increase in the expression of costimulatory factors CD80 and CD40 on APCs in tumor-draining lymph nodes (Figure 8C, 8D). In addition to changes consistent with an increased cytotoxic T cell response, the frequency of activated NK cells in the tumor, spleen, and blood, was significantly increased in response to combination treatment. Interestingly, low-dose CY and the combination treatment had similar effects on splenic NK cell activation, but this change was not reflected in the TME and only reached statistical significance in response to combination treatment in the tumor (Supplementary Figure 7A).

The ultimate goal of most anti-tumor immune therapies in humans is to maximize the cytotoxic T cell response to tumor cells. To confirm that the anti-tumor activity generated by combination therapy was based on cytotoxic T cells, a CD8-depletion experiment was performed. Depletion of CD8+ cells abrogated the anti-tumor response generated by the combination of intratumoral SD-101 plus low-dose CY (Figure 8E). Depletion of CD8+ T cells was confirmed in blood of treated mice (Supplementary Figure 7B). Furthermore, when mice that had been treated with combination SD-101 and low-dose CY and had cleared the primary tumors (from Figure 2D) were rechallenged with the same tumor type after 86 days, these mice were able to fully control tumor growth without any further treatment (Supplementary Figure 7C). This suggests the combination therapy induced long-term anti-tumor immune memory and that the mice were cured.

## DISCUSSION

Despite recent breakthroughs in immune therapy of cancer, it is appreciated that combination therapies that increase efficacy will be needed to treat patients whose tumors do not respond to current and emerging standards of care. SD-101 has been combined successfully with PD-1 blockade to achieve increased efficacy with minimal added toxicity [7, 8], yet not all patients respond to this doublet. This study evaluated the potential of adding low-dose chemotherapies to therapeutic regimens, as these generic drugs are well tolerated at low-dose and have been shown to have immune-modulatory actions that would be predicted to be complementary to those of SD-101. Anti-PD-1 therapy is already established as the standard of care for many tumor types and seemingly requires the presence of T cells that can be reinvigorated [39, 40]. Thus, we focused these pre-clinical studies on the potential of low-dose chemotherapy to enhance the ability of SD-101 to stimulate anti-tumor T cell responses. We surveyed several low-dose chemotherapies (data not shown) and identified low-dose cyclophosphamide as the most promising candidate for combination with SD-101.

The combination of intratumoral SD-101 plus systemic low-dose metronomic cyclophosphamide led to rapid anti-tumor responses mediated by CD8+ T cells. These robust anti-tumor responses reflected the generation of a systemic anti-tumor immune response, where both injected and non-injected tumors were rejected and cured mice were resistant to tumor cell rechallenge. The anti-tumor effects of the combination were observed in multiple tumor models and in tumors in different anatomical locations (sub-cutaneous and lung). Notably, substantial anti-tumor activity was observed even in very large tumors that were unresponsive to treatment with either single agent.

To understand the mechanistic basis for the strong systemic anti-tumor responses with the combination, we examined global gene expression changes and characterized the immune infiltrate in the TME at multiple times during the course of treatment. The combination of low-dose metronomic cyclophosphamide and intratumoral SD-101 strongly potentiated the innate immune components early, and this, together with a relief of immunosuppression in the TME, ultimately led to the generation of an effective systemic antitumor CD8+ T cell response, as well as immunologic memory.

Nanostring™ analysis of gene expression over the time course of treatment with SD-101, low-dose CY, or the combination, showed clear contrasts between treatment with the combination and either single agent. Gene expression changes overall with the combination were much more profound than with either monotherapy, and showed rapid and durable activation of immune functions that help explain its substantial therapeutic benefit. Potentiation of innate immunity was apparent immediately, showing impact on the interferon pathway and antigen processing after the first administration of the combination. Combination treatment resulted in substantial early interferon induction, revealing complementarity of SD-101 and low-dose CY in interferon regulation. We observed that both low-dose CY and SD-101 monotherapies induced interferon-regulated genes, as has been previously shown [5, 18, 41]. There were both common (e.g. *Gbp5, Ifi44, Ifih1, Ifit1, Ifit3, and Irf7*) and unique genes that CY (*Eomes, Ifi44l, Irf8, Irgm2, Nlrc5*) and SD-101 (*Ccr7, Ifi35, Ifit2, Nos2, Runx3, Sh2d1b1* and *Tbk1*) up-regulated in response to the respective monotherapies. The interferon pathway was further activated by the combination, with up-regulation of 25 out of 34 genes in the interferon gene set (non-injected site) (Supplementary Figure 3E). Using expression of fifteen IFN-inducible genes to define an IFNα gene signature, combination treatment resulted in a significantly elevated IFNα gene signature at d19 (Supplementary Figure 3F).

Innate responses were apparent at the earliest time points and adaptive responses developed most clearly at later time points. We observed significant changes in pathway and cell type scores at d19, after a lead-in dose of CY followed by a single combination treatment of CY and SD-101 (Figure 4E and 4F). Low-dose CY alone increased the antigen processing and MHC signatures modestly, but combination treatment increased these to a significant degree. For example, there was higher expression of *Tap1*, *Tap2* and *Tapbp* in the low-dose CY monotherapy group compared to control, and a further upregulation of these genes in the combination treatment. *Tap1*, *Tap2* and *Tapbp* are involved in the transport of antigens from the cytoplasm to the endoplasmic reticulum for association with MHC class I molecules. Additionally, Nanostring™ analysis of the combination showed an increase in gene signatures associated with heightened dendritic cell function. In both cases, these pathway scores trended higher in the combination than in response to either single agent. Our functional analysis of antigen processing *in vivo*, using DQ-OVA (Figure 6C), also revealed an increase in antigen processing in combination-treated tumors. This was likely further potentiated by the induction of immunogenic cell death, as indicated by increased tumor cell surface calreticulin (Figure 6D). This suggests that the combination of these two agents created an improved environment in the tumor for antigens to be optimally presented and prime T cells. Increased priming was also supported by our observation of increased T cell frequency and increased expression of costimulatory molecules CD40 and CD86 on APCs in the tumor-draining lymph nodes. While increases in pathways corresponding to immune activation were also induced by the monotherapies to some degree, combination therapy resulted in more significant immune activation overall and, unlike the monotherapies, these effects were sustained at least one week following the stoppage of treatment. Thus, not only did low-dose CY augment the earliest immune-modulatory effects of SD-101, but it enabled these effects to be sustained. Collectively, the increased interferon induction and antigen processing in the combination are likely potential components of the mechanism that conferred the increased systemic CD8+ T cell dependent antitumor response.

Cyclophosphamide has been described, in some studies, to expand immunosuppressive myeloid populations [20, 27–29], although direct evidence of the overall impact of these CY-induced cells in the TME is incomplete and at times contradictory [20]. We did not observe an increase in PMN-MDSCs at the low dose of 40 mg/kg CY (Figure 1E). We did observe an increase in activated Ly6C+ CD11b+ monocytes in the tumor in response to low-dose CY (Figure 1G). Interestingly, a subset of the Ly6C+ CD11b+ population is upregulated in response to another chemotherapy, oxaliplatin, and was shown to exert APC functions [42]. Furthermore, a study of B-class CpG ODN with CY showed that cells within the subset of CD11b+Gr^dim^ cells (Ly6G-), which are mainly Ly6C+, are tumoricidal [43]. We showed that low-dose CY induces an increase in the expression of MHCII in CD11b+ Ly6C+ monocytes, and combination therapy increases MHCII, CD40, PDL1, and TNF in this subset, all of which are associated with activation of monocytes, and/or response to inflammatory cytokines [44–46]. However, we did not test directly whether this myeloid subset contributed to the anti-tumor effects.

In addition to antigen processing and MHC increases, early activation of the complement pathway was also observed in both the injected and non-injected tumors (Figure 4D & Supplementary Figure 3A). Both low-dose CY (*C1ra, C1s1, C3, C4b, Cd55, Cfb* and *Serping1*) and SD-101 (*C2, C3, Cfb, Cfd* and *Serping1*) alone activated select complement components (Supplementary Figure 3D). The impact of CY on the complement pathway is consistent with another study in which increased expression of complement components by metronomic CY in tumor xenografts was observed [41], although this study used a higher dose of CY. In the non-injected tumor, combination treatment resulted in up-regulation of 15 out of 18 genes in the complement pathway including anaphylatoxin receptors (*C3ar1, C5ar1*), complement regulators (*Cfh, Cfp*), and further up-regulation of *C3*, which is a central node in the complement cascade on which both classical and alternative pathways converge (Supplementary Figure 3D). It is unknown at this time whether this impact on the complement pathway contributes to the efficacy observed.

The balance of immunosuppressive, pro-tumor versus anti-tumor myeloid cells in the TME is pivotal to determining whether effector immune cells can access and attack the tumor for effective immunotherapy [1]. Here, both gene expression and flow cytometry immune profiling data provide a mechanistic explanation for the efficacy derived from relieving immunosuppression in the TME. We showed that the combination treatment reduces CD206, typically associated with pro-tumor M2-like macrophages, while increasing factors associated with M1-like macrophages (Figure 7A, 7B and Supplementary Figure 3G). We also confirmed, as has been repeatedly demonstrated in the literature[15, 16, 21], that cyclophosphamide potently depletes Tregs, even at low doses (Figure 1A). The combination of these agents results in potent Treg depletion (Figure 8A). Teff/ Treg ratios are, in turn, increased to mediate an optimal T cell response (Figure 8B).

Consistent with a temporal progression from innate stimulation to adaptive immune responses, we observed T cell and cytotoxic cell scores progressively increasing at later time points (Figure 4F). Importantly, effector cell functions were maintained in the combination even a week after stopping treatment, unlike the respective monotherapies. Ultimately, our data suggest that low-dose CY in combination with SD-101 provides an overall stimulatory environment that promotes CD8+ T cell mediated antitumor responses and immunologic memory, simultaneously increasing antigen processing and priming of T cells, while relieving TME immunosuppression.

There have been a few reports showing that combination of a TLR9 agonist CpG-ODN with CY results in anti-tumor effects in mouse tumor models [43, 47, 48]. These studies used B-Class CpG-ODNs, which stimulate much lower type 1 IFN responses than C-class CpG-ODN, the class used in our study [49]. In the study by Manrique and colleagues, the combination of B-Class CpG-ODN with CY did result in tumor regression in mice, however immunologic memory was not generated, and a higher dose of CY was needed for consistent efficacy [43]. Consistent with our results, Jordan and Waxman observed anti-tumor effects in an immunogenic glioma model, using low-dose CY plus a B-class CpG-ODN, and showed an increase in intratumoral CD11b+ F4/80+ myeloid cells, NK cells, DCs and T cells, as well as immunologic memory [47]. The detailed mechanistic analysis we presented here, including early kinetics of gene expression changes by the respective monotherapies, as well as the combination, add considerable insight into understanding the potential therapeutic value of combining CpG-ODNs and low-dose CY in treating tumors. We extend the understanding of mechanisms of action of the combination, showing a shift in the TME towards M1-like TAM phenotypes and increased antigen presentation, which may contribute to the increased efficacy.

An ongoing Phase 1b/2 clinical trial (ClinicalTrials.gov Identifier NCT02521870) of SD-101 in combination with pembrolizumab is yielding a high rate of response in melanoma patients [7, 8]. Although immune checkpoint blockade therapies such as pembrolizumab have thus far improved survival in subsets of patients, it is apparent that some cancer types respond better than others, and there is still significant unmet need for improved immunotherapy to reach a greater patient population. Our data indicates that low-dose metronomic CY in combination with intratumoral SD-101 results in increased innate and adaptive anti-tumor immune responses to optimize CD8+ T cell killing of tumor cells and immunologic memory. These results provide the basis for clinical evaluation of the addition of low-dose CY to treatment regimens that include a TLR9 agonist. Taken together, the intratumoral SD-101 plus low-dose CY combination may complement existing checkpoint blockade therapies in patients to improve efficacy in the clinic and extend the benefits of immunotherapy to more patients.

## MATERIALS AND METHODS

### Mice, cell lines and reagents

Six to eight week old female BALB/c mice were purchased from Envigo. Mice were maintained in Dynavax’s or MuriGenics’ vivariums. All animal protocols were approved by the Institutional Animal Care and Use Committees (IACUC) of the respective institutions. CT26 colon carcinoma cells (CRL-2639) and 4T1 mammary carcinoma cells (CRL-2539) were purchased from ATCC and authenticated by ATCC using COI analysis. Cell lines were negative for mycoplasma testing and were passaged twice before using for *in vivo* experiments. The CpG-C oligodeoxynucleotide SD-101 was synthesized and purified by standard techniques as described previously [49]. Cyclophosphamide (CY) was obtained from Sigma. Anti-mouse CD8 (YTS 169.4, BE0117) for the *in vivo* depletion study was purchased from BioXCell.

### Mouse tumor models and treatments

For the syngeneic tumor mouse models, CT26 cells (8x10^4^) or 4T1 cells (1.2x10^4^) were inoculated subcutaneously in both flanks of BALB/c mice. Tumor growth was monitored twice weekly using digital calipers and recorded with the Studylog system. Tumor volume was calculated using the following formula: volume= ½ X length X (width)^2^. When tumors reached an average volume of 200 mm^3^, mice were randomized into (1) control, (2) SD-101, (3) CY or (4) SD-101+ CY groups and CY treatment began. Low-dose CY (40 mg/kg body weight) was given intraperitoneally twice weekly to mice of the CY and combination groups. SD-101 treatment (50μg/ 100 μL saline) was injected into the right flank tumor (injected tumor) of the mice in the SD-101 and combination groups twice weekly. SD-101 treatment began 2-4 days after the first CY treatment, when average tumor volume reached 300-400 mm^3^. Control mice received saline only. For mechanistic studies, mice received 1.5- 2.5 weeks treatment. For efficacy and long-term survival studies, mice received 2-3 weeks of treatment. Treatment schedules are indicated in the experimental schema within figures. For the rechallenge study, surviving mice from the survival study were rechallenged with a second implantation of CT26 at d86 post first CT26 implantation and tumor growth was monitored.

To establish CT26 pulmonary metastasis, CT26 intravenous lung metastasis models were used [6]. In the CT26 i.v. model, CT26 cells (1 x10^4^) were injected intravenously (i.v.) into the mice to induce dissemination into the lungs. CY treatment (16 mg/kg) was given orally (p.o.) 5 times weekly. SD-101 (10 μg/50 μl saline) was given intranasally (i.n.) biweekly. Control mice received saline (i.n.) only. CY and SD-101 treatment began at d14 and d17 post CT26 inoculation, respectively, for three weeks. Survival was monitored.

### *In vivo* CD8 depletion

Anti-CD8 (YTS 169.4, BE0117, Bio XCell) *in vivo* depletion antibodies were given i.p. at 250 μg per injection one day prior to CY treatment and twice weekly until the end of the experiment. CD8 depletion efficiency was confirmed by flow cytometric analyses of CD45+ CD3+ CD8+ cells (Biolegend) in the blood of the experimental mice (Supplementary Figure 7B).

### *In vivo* antigen uptake and processing study

CT26 cells (1x10^5^) were inoculated subcutaneously in both flanks of BALB/c mice. CY treatment (40 mg/kg) was given intraperitoneally when average tumor volume reached ~ 200 mm^3^ at day 14 and the second dose was given at day 18. One dose of SD-101 (50μg/ 100 μL saline) was injected into the right flank tumor (injected-tumor) of the mice 4 days after the first CY treatment at day 18. DQ-OVA (50 μg) was injected into the right flank of the mice at day 18 and 20 as shown in Figure 6B. Tumors were harvested 24 hours after the last DQ-OVA injection. DQ-OVA-derived fluorescence-positive cells (FITC and TexasRed channels) were evaluated in the gated CD3-CD19-SiglecF-DX5-CD45+ immune cells by flow cytometry. Data were acquired using LSRII Flow Cytometer (BD Biosciences) and analyzed using FlowJo software (BD Biosciences).

### Isolation of leukocytes from tumor-bearing mice and flow cytometry

Tumors, spleen and tumor draining lymph nodes were collected and processed using the gentleMACS dissociator (Miltenyi Biotec) as previously described [5]. Briefly, cells were enzymatically digested with 50 mg/mL of collagenase 4 (Sigma) and 20 mg/mL of DNase I (Sigma) in wash medium for 20 minutes at 37ºC in 5% CO_2_. The digested samples were filtered through 70 μM strainer and washed. Leukocytes from blood were isolated using 2% dextran sulfate (Sigma). Cells were collected after 20 min of sedimentation, lysed with RBC lysing buffer and washed. For live/ dead discrimination, LIVE/DEAD Fixable Yellow Dead Cell Stain kit (Thermo Fisher Scientific) was used. Single cell suspensions were blocked with mouse Fc-Block (BD Biosciences) and then labeled with several antibody panels staining for CD45, CD3, CD4, CD8, CD11b, DX5, PDL1, MHCII, CD107α, Ly6-G, Ly6-C, CD80, CD40, CD206, CD19, Siglec-F, CD326 (BioLegend and eBioscience) and calreticulin (Abcam). For intracellular staining, cells were further permeabilized using Transcription Factor Buffer set kit (BD Biosciences) and stained for Foxp3, T-bet and TNFα. Data were acquired using LSRII Flow Cytometer (BD Biosciences) and analyzed using FlowJo software (BD Biosciences).

### NanoString™ gene expression profiling

Total RNA from tumors was extracted using RNeasy kit (Qiagen) and hybridized with the NanoString nCounter PanCancer Immune profiling mouse panel code set (NanoString™, Seattle, WA). The differential mRNA expression of 770 genes (730 immune genes and 40 housekeeping genes) was quantified using the nCounter Digital Analyzer at the Core Diagnostics, Inc. After zero-expression genes were removed, a total of 582 genes were included in the group comparison. NanoString results were produced from RCC files using nSolver software (version3.0). The QC, normalization, differential expression, functional pathways and cell types score analyses were performed using the nSolver advanced analysis module according to guidance given by the manufacturers.

### Quantitative real-time PCR

RNA was extracted from whole tumors using an RNeasy Mini kit (Qiagen). cDNA was generated using Superscript III reverse transcriptase (Thermo Fisher). Quantitative Real-Time PCR was carried out using the SYBR® Green master mix (Thermo Fisher) with the real-time PCR primers (Eurofins Genomics). Primer sequences are listed in Supplementary Table 1. Expression levels were normalized to ubiquitin using delta-delta Ct methods.

### Statistical analysis

All statistical analyses were performed using Prism software v5 (GraphPad Software). A two-tailed unpaired Mann-Whitney U test was used to compare two groups. Multiple comparisons were performed using the one-way ANOVA test with the Tukey *post hoc* test. For tumor volumes collected over all time points, two-way repeated measures ANOVA was used to compare different groups, using Tukey’s multiple comparison test. Survival curves were plotted using the Kaplan-Meier method and compared using the log-rank test. Correlations between tumor volumes and gene expression levels were analyzed using a Spearman rank correlation test. P values lower than 0.05 were considered statistically significant (^*^, P<0.05; ^**^, P<0.01; ^***^, P<0.001; and ^****^, P<0.0001).

## SUPPLEMENTARY MATERIALS FIGURES





## Abbreviations

TLR9: toll-like receptor 9
CY: cyclophosphamide
CpG-ODN: CpG oligodeoxynucleotides
pDC: plasmacytoid dendritic cells
DC: dendritic cells
NK: natural killer cells
Treg: regulatory T-cells
CD4+Tconv: conventional CD4 T cells
MDSC: myeloid-derived suppressor cells
PMN-MDSC: polymorphonuclear myeloid derived suppressor cells
MO-MDSC: monocytic myeloid-derived suppressor cells
Teff: effector T cells
TAM: tumor-associated macrophages
DE: differentially expressed
s.c: subcutaneous
i.t: intratumoral
i.v: intravenous
p.o.: per os
i.p: intraperitoneal
i.n: intranasal
I: injected
NI: non-injected
tdLN: tumor draining lymph nodes
TME: tumor microenvironment
PD-1: programmed death-1
PD-L1: programmed death-ligand 1
IFN: interferon
TNF: tumor necrosis factor.

## References

[1] Chen DS , Mellman I. Elements of cancer immunity and the cancer-immune set point. Nature. 2017; 541: 321–30. 10.1038/nature21349. 28102259

[2] Schumacher TN , Schreiber RD. Neoantigens in cancer immunotherapy. Science. 2015; 348: 69–74. 10.1126/science.aaa4971. 25838375

[3] Pons-Tostivint E , Latouche A , Vaflard P , Ricci F , Loirat D , Hescot S , Sablin MP , Rouzier R , Kamal M , Morel C , Lecerf C , Servois V , Paoletti X , et al Comparative Analysis of Durable Responses on Immune Checkpoint Inhibitors Versus Other Systemic Therapies: A Pooled Analysis of Phase III Trials. JCO Precis Oncol. 2019; 35:1–10. 10.1200/po.18.00114. 35100670

[4] Sharma P , Allison JP. The future of immune checkpoint therapy. Science. 2015; 348: 56–61. 10.1126/science.aaa8172. 25838373

[5] Wang S , Campos J , Gallotta M , Gong M , Crain C , Naik E , Coffman RL , Guiducci C. Intratumoral injection of a CpG oligonucleotide reverts resistance to PD-1 blockade by expanding multifunctional CD8+ T cells. Proc Natl Acad Sci U S A. 2016; 113: E7240–E9. 10.1073/pnas.1608555113. 27799536 PMC5135381

[6] Gallotta M , Assi H , Degagne E , Kannan SK , Coffman RL , Guiducci C. Inhaled TLR9 Agonist Renders Lung Tumors Permissive to PD-1 Blockade by Promoting Optimal CD4+ and CD8+ T cell Interplay. Cancer Res. 2018; 78: 4943–56. 10.1158/0008-5472.CAN-18-0729. 29945961

[7] Ribas A , Medina T , Kummar S , Amin A , Kalbasi A , Drabick JJ , Barve M , Daniels GA , Wong DJ , Schmidt EV , Candia AF , Coffman RL , Leung ACF , et al. SD-101 in Combination with Pembrolizumab in Advanced Melanoma: Results of a Phase Ib, Multicenter Study. Cancer Discov. 2018; 8: 1250–7. 10.1158/2159-8290.CD-18-0280. 30154193 PMC6719557

[8] Milhem MM , Long GV , Hoimes CJ , Amin A , Lao CD , Conry RM , Hunt J , Daniels GA , Almubarak M , Shaheen MF , Medina TM , Barve MA , Bishnoi SK , et al Phase 1b/2, open label, multicenter, study of the combination of SD-101 and pembrolizumab in patients with advanced melanoma who are naïve to anti-PD-1 therapy. J Clin Oncol. 2019; 9534 10.1200/JCO.2019.37.15_suppl.9534.

[9] Guiducci C , Ott G , Chan JH , Damon E , Calacsan C , Matray T , Lee KD , Coffman RL , Barrat FJ. Properties regulating the nature of the plasmacytoid dendritic cell response to Toll-like receptor 9 activation. J Exp Med. 2006; 203: 1999–2008. 10.1084/jem.20060401. 16864658 PMC2118381

[10] Zitvogel L , Galluzzi L , Kepp O , Smyth MJ , Kroemer G. Type I interferons in anticancer immunity. Nat Rev Immunol. 2015; 15: 405–14. 10.1038/nri3845. 26027717

[11] Corrales L , Matson V , Flood B , Spranger S , Gajewski TF. Innate immune signaling and regulation in cancer immunotherapy. Cell Res. 2017; 27: 96–108. 10.1038/cr.2016.149. 27981969 PMC5223230

[12] Colleoni M , Rocca A , Sandri MT , Zorzino L , Masci G , Nolè F , Peruzzotti G , Robertson C , Orlando L , Cinieri S , de BF , Viale G , Goldhirsch A. Low-dose oral methotrexate and cyclophosphamide in metastatic breast cancer: antitumor activity and correlation with vascular endothelial growth factor levels. Ann Oncol. 2002; 13: 73–80. 10.1093/annonc/mdf013. 11863115

[13] Glode LM , Barqawi A , Crighton F , Crawford ED , Kerbel R. Metronomic therapy with cyclophosphamide and dexamethasone for prostate carcinoma. Cancer. 2003; 98: 1643–8. 10.1002/cncr.11713. 14534880

[14] Kerbel RS , Kamen BA. The anti-angiogenic basis of metronomic chemotherapy. Nat Rev Cancer. 2004; 4: 423–36. 10.1038/nrc1369. 15170445

[15] Ghiringhelli F , Menard C , Puig PE , Ladoire S , Roux S , Martin F , Solary E , Le Cesne A , Zitvogel L , Chauffert B. Metronomic cyclophosphamide regimen selectively depletes CD4+CD25+ regulatory T cells and restores T and NK effector functions in end stage cancer patients. Cancer Immunol Immunother. 2007; 56: 641–8. 10.1007/s00262-006-0225-8. 16960692 PMC11030569

[16] Berd D , Mastrangelo MJ. Effect of low dose cyclophosphamide on the immune system of cancer patients: reduction of T-suppressor function without depletion of the CD8+ subset. Cancer Res. 1987; 47: 3317–21. 2953413

[17] Ge Y , Domschke C , Stoiber N , Schott S , Heil J , Rom J , Blumenstein M , Thum J , Sohn C , Schneeweiss A , Beckhove P , Schuetz F. Metronomic cyclophosphamide treatment in metastasized breast cancer patients: immunological effects and clinical outcome. Cancer Immunol Immunother. 2012; 61: 353–62. 10.1007/s00262-011-1106-3. 21915801 PMC11028651

[18] Moschella F , Torelli GF , Valentini M , Urbani F , Buccione C , Petrucci MT , Natalino F , Belardelli F , Foa R , Proietti E. Cyclophosphamide induces a type I interferon-associated sterile inflammatory response signature in cancer patients’ blood cells: implications for cancer chemoimmunotherapy. Clin Cancer Res. 2013; 19: 4249–61. 10.1158/1078-0432.CCR-12-3666. 23759676

[19] Wu J , Waxman DJ. Immunogenic chemotherapy: Dose and schedule dependence and combination with immunotherapy. Cancer Lett. 2018; 419: 210–21. 10.1016/j.canlet.2018.01.050. 29414305 PMC5818299

[20] Bracci L , Schiavoni G , Sistigu A , Belardelli F. Immune-based mechanisms of cytotoxic chemotherapy: implications for the design of novel and rationale-based combined treatments against cancer. Cell Death Differ. 2014; 21: 15–25. 10.1038/cdd.2013.67. 23787994 PMC3857622

[21] Ahlmann M , Hempel G. The effect of cyclophosphamide on the immune system: implications for clinical cancer therapy. Cancer Chemother Pharmacol. 2016; 78: 661–71. 10.1007/s00280-016-3152-1. 27646791

[22] Ding ZC , Munn DH , Zhou G. Chemotherapy-induced myeloid suppressor cells and antitumor immunity: The Janus face of chemotherapy in immunomodulation. Oncoimmunology. 2014; 3: e954471. 10.4161/21624011.2014.954471. 25610747 PMC4292425

[23] Salem ML , Al-Khami AA , El-Naggar SA , Diaz-Montero CM , Chen Y , Cole DJ. Cyclophosphamide induces dynamic alterations in the host microenvironments resulting in a Flt3 ligand-dependent expansion of dendritic cells. J Immunol. 2010; 184: 1737–47. 10.4049/jimmunol.0902309. 20083664 PMC3066076

[24] Liu P , Jaffar J , Hellstrom I , Hellstrom KE. Administration of cyclophosphamide changes the immune profile of tumor-bearing mice. J Immunother. 2010; 33: 53–9. 10.1097/CJI.0b013e3181b56af4. 19952956 PMC2811714

[25] Scurr M , Pembroke T , Bloom A , Roberts D , Thomson A , Smart K , Bridgeman H , Adams R , Brewster A , Jones R , Gwynne S , Blount D , Harrop R , et al. Low-Dose Cyclophosphamide Induces Antitumor T-Cell Responses, which Associate with Survival in Metastatic Colorectal Cancer. Clin Cancer Res. 2017; 23: 6771–80. 10.1158/1078-0432.CCR-17-0895. 28855352 PMC5769815

[26] Lutsiak ME , Semnani RT , De Pascalis R , Kashmiri SV , Schlom J , Sabzevari H. Inhibition of CD4(+)25+ T regulatory cell function implicated in enhanced immune response by low-dose cyclophosphamide. Blood. 2005; 105: 2862–8. 10.1182/blood-2004-06-2410. 15591121

[27] Angulo I , de las Heras FG , García-Bustos JF , Gargallo D , Muñoz-Fernández MA , Fresno M . Nitric oxide-producing CD11b(+)Ly-6G(Gr-1)(+)CD31(ER-MP12)(+) cells in the spleen of cyclophosphamide-treated mice: implications for T-cell responses in immunosuppressed mice. Blood. 2000; 95: 212–20. 10607705

[28] Ding ZC , Lu X , Yu M , Lemos H , Huang L , Chandler P , Liu K , Walters M , Krasinski A , Mack M , Blazar BR , Mellor AL , Munn DH , et al. Immunosuppressive myeloid cells induced by chemotherapy attenuate antitumor CD4+ T-cell responses through the PD-1-PD-L1 axis. Cancer Res. 2014; 74: 3441–53. 10.1158/0008-5472.CAN-13-3596. 24780756 PMC4079842

[29] Mikyskova R , Indrova M , Pollakova V , Bieblova J , Simova J , Reinis M. Cyclophosphamide-induced myeloid-derived suppressor cell population is immunosuppressive but not identical to myeloid-derived suppressor cells induced by growing TC-1 tumors. J Immunother. 2012; 35: 374–84. 10.1097/CJI.0b013e318255585a. 22576342

[30] Kratochvill F , Neale G , Haverkamp JM , Van de Velde LA , Smith AM , Kawauchi D , McEvoy J , Roussel MF , Dyer MA , Qualls JE , Murray PJ. TNF Counterbalances the Emergence of M2 Tumor Macrophages. Cell Rep. 2015; 12: 1902–14. 10.1016/j.celrep.2015.08.033. 26365184 PMC4581986

[31] Galluzzi L , Buque A , Kepp O , Zitvogel L , Kroemer G. Immunogenic cell death in cancer and infectious disease. Nat Rev Immunol. 2017; 17: 97–111. 10.1038/nri.2016.107. 27748397

[32] Gabrilovich DI , Ostrand-Rosenberg S , Bronte V. Coordinated regulation of myeloid cells by tumours. Nat Rev Immunol. 2012; 12: 253–68. 10.1038/nri3175. 22437938 PMC3587148

[33] Cassetta L , Pollard JW. Targeting macrophages: therapeutic approaches in cancer. Nat Rev Drug Discov. 2018; 17: 887–904. 10.1038/nrd.2018.169. 30361552

[34] Ruffell B , Coussens LM . Macrophages and therapeutic resistance in cancer. Cancer Cell. 2015; 27:462-72. 10.1016/j.ccell.2015.02.015. 25858805 PMC4400235

[35] Guiducci C , Vicari AP , Sangaletti S , Trinchieri G , Colombo MP. Redirecting *in vivo* elicited tumor infiltrating macrophages and dendritic cells towards tumor rejection. Cancer Res. 2005; 65: 3437–46. 10.1158/0008-5472.CAN-04-4262. 15833879

[36] Sato-Kaneko F , Yao S , Ahmadi A , Zhang SS , Hosoya T , Kaneda MM , Varner JA , Pu M , Messer KS , Guiducci C , Coffman RL , Kitaura K , Matsutani T , et al. Combination immunotherapy with TLR agonists and checkpoint inhibitors suppresses head and neck cancer. JCI Insight. 2017; 2. 10.1172/jci.insight.93397. 28931759 PMC5621908

[37] Shevach EM. Mechanisms of foxp3+ T regulatory cell-mediated suppression. Immunity. 2009; 30: 636–45. 10.1016/j.immuni.2009.04.010. 19464986

[38] Gao Q , Qiu SJ , Fan J , Zhou J , Wang XY , Xiao YS , Xu Y , Li YW , Tang ZY. Intratumoral balance of regulatory and cytotoxic T cells is associated with prognosis of hepatocellular carcinoma after resection. J Clin Oncol. 2007; 25: 2586–93. 10.1200/JCO.2006.09.4565. 17577038

[39] Kurtulus S , Madi A , Escobar G , Klapholz M , Nyman J , Christian E , Pawlak M , Dionne D , Xia J , Rozenblatt-Rosen O , Kuchroo VK , Regev A , Anderson AC. Checkpoint Blockade Immunotherapy Induces Dynamic Changes in PD-1(-)CD8(+) Tumor-Infiltrating T Cells. Immunity. 2019; 50: 181–94.e6. 10.1016/j.immuni.2018.11.014. 30635236 PMC6336113

[40] Siddiqui I , Schaeuble K , Chennupati V , Fuertes Marraco SA , Calderon-Copete S , Pais Ferreira D , Carmona SJ , Scarpellino L , Gfeller D , Pradervand S , Luther SA , Speiser DE , Held W. Intratumoral Tcf1(+)PD-1(+)CD8(+) T Cells with Stem-like Properties Promote Tumor Control in Response to Vaccination and Checkpoint Blockade Immunotherapy. Immunity. 2019; 50: 195–211.e10. 10.1016/j.immuni.2018.12.021. 30635237

[41] Doloff JC , Waxman DJ. Transcriptional profiling provides insights into metronomic cyclophosphamide-activated, innate immune-dependent regression of brain tumor xenografts. BMC Cancer. 2015; 15: 375. 10.1186/s12885-015-1358-y. 25952672 PMC4523019

[42] Ma Y , Adjemian S , Mattarollo SR , Yamazaki T , Aymeric L , Yang H , Portela Catani JP , Hannani D , Duret H , Steegh K , Martins I , Schlemmer F , Michaud M , et al. Anticancer chemotherapy-induced intratumoral recruitment and differentiation of antigen-presenting cells. Immunity. 2013; 38: 729–41. 10.1016/j.immuni.2013.03.003. 23562161

[43] Manrique SZ , Dominguez AL , Mirza N , Spencer CD , Bradley JM , Finke JH , Lee JJ , Pease LR , Gendler SJ , Cohen PA. Definitive activation of endogenous antitumor immunity by repetitive cycles of cyclophosphamide with interspersed Toll-like receptor agonists. Oncotarget. 2016; 7: 42919–42. 10.18632/oncotarget.10190. 27341020 PMC5189997

[44] Jakubzick CV , Randolph GJ , Henson PM. Monocyte differentiation and antigen-presenting functions. Nat Rev Immunol. 2017; 17: 349–62. 10.1038/nri.2017.28. 28436425

[45] Alderson MR , Armitage RJ , Tough TW , Strockbine L , Fanslow WC , Spriggs MK. CD40 expression by human monocytes: regulation by cytokines and activation of monocytes by the ligand for CD40. J Exp Med. 1993; 178: 669–74. 10.1084/jem.178.2.669. 7688031 PMC2191134

[46] Sun C , Mezzadra R , Schumacher TN. Regulation and Function of the PD-L1 Checkpoint. Immunity. 2018; 48: 434–52. 10.1016/j.immuni.2018.03.014. 29562194 PMC7116507

[47] Wu J , Waxman DJ. Metronomic cyclophosphamide eradicates large implanted GL261 gliomas by activating antitumor Cd8(+) T-cell responses and immune memory. Oncoimmunology. 2015; 4: e1005521. 10.1080/2162402X.2015.1005521. 26137402 PMC4485826

[48] Gershan JA , Barr KM , Weber JJ , Jing W , Johnson BD. Immune modulating effects of cyclophosphamide and treatment with tumor lysate/CpG synergize to eliminate murine neuroblastoma. J Immunother Cancer. 2015; 3: 24. 10.1186/s40425-015-0071-3. 26082836 PMC4469315

[49] Marshall JD , Fearon KL , Higgins D , Hessel EM , Kanzler H , Abbate C , Yee P , Gregorio J , Cruz TD , Lizcano JO , Zolotorev A , McClure HM , Brasky KM , et al. Superior activity of the type C class of ISS *in vitro* and *in vivo* across multiple species. DNA Cell Biol. 2005; 24: 63–72. 10.1089/dna.2005.24.63. 15699627

