# Supplementary material for: Low-dose metronomic cyclophosphamide complements the actions of an intratumoral C-class CpG TLR9 agonist to potentiate innate immunity and drive potent T cell-mediated anti-tumor responses
Source: Oncotarget. 2019 Dec 31;10(68):7220–37. doi: 10.18632/oncotarget.27322 (PMC6944447; doi:10.18632/oncotarget.27322)
Supplement: Supplementary file 1 [file oncotarget-10-7220-s001.pdf]

Low-dose metronomic cyclophosphamide complements the actions of an intratumoral C-class CpG TLR9 agonist to potentiate innate immunity and drive potent T cell-mediated anti-tumor responses

SUPPLEMENTARY MATERIALS

Supplementary Table 1: DE genes in combination treatment only, but not in monotherapy treatments

See Supplementary Table 1

Supplementary Table 2: Primers

IFN-gamma	F	TCAAGTGGCATAGATGTGGAAGAA
	R	TGGCTCTGCAGGATTTTCATG
TNF-alpha	F	GCCACCACGCTCTTCTGTCT
	R	GGTCTGGGCCATAGAACTGATG
CXCL10	F	GACGGTCCGCTGCAACTG
	R	GCTTCCCTATGGCCCTCATT
CXCL9	F	TGCACGATGCTCCTGCA
	R	AGGTCTTTGAGGGATTTGTAGTGG
CCL5	F	GCAAGTGCTCCAATCTTGCA
	R	CTTCTCTGGGTTGGCACACA
CD8 beta	F	TCAAGACGGCCCTTTCTCAGT
	R	ACCGTCGCGCAGAAGTAGA
CD80	F	TGAAGACCGAATCTACTGGCAAA
	R	AAGAGAGTAGGTAGTGTTGTCATATAAAGTCC
NOS-2	F	CCGATTTAGAGTCTTGGTGAAAGTG
	R	TGACCCGTGAAGCCATGA
ICAM-1	F	TCGGAAGGGAGCCAAGTAACT
	R	CGACGCCGCTCAGAAGAA
IL-1b	F	GACGGCACACCCACCCT
	R	AAACCGTTTTTCCATCTTCTTCTTT
IL-6	F	ACACATGTTCTCTGGGAAATCGT
	R	AAGTGCATCATCGTTGTTCATACA
IL-11	F	TGCTGACAAGGCTTCGAGTAGA
	R	TTCCAGTCGGGCTTGCA
IL-18	F	CACAACAAGATGGAGTTTGAATCTTC
	R	AGAATGAGTTTGAAAGCATCATCTTC
COX-2	F	CCAGCACTTCACCCATCAGTT
	R	AAGGCGCAGTTTATGTTGTCTGT
ubiquitin	F	TGGCTATTAATTATTCGGTCTGCAT
	R	GCAAGTGGCTAGAGTGCAGAGTAA

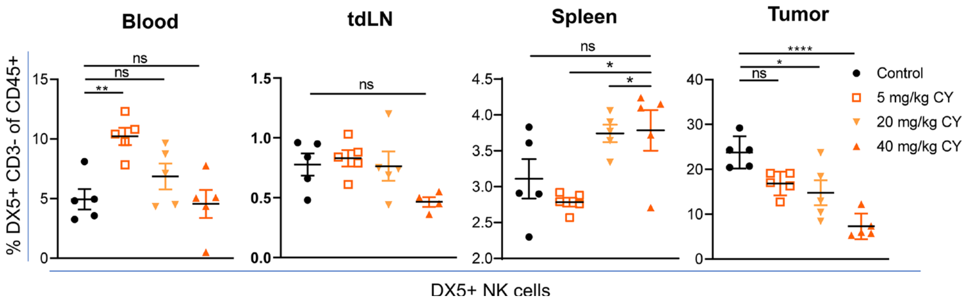

Supplementary Figure 1: Frequency of DX5+ NK of total CD45+ in response to increasing doses CY (i.p.). Data reflect two weeks of CY treatment, twice weekly (i.p.). Graphs represent one independent experiment. Data are the mean ± SEM, n=5/ group. * indicates P ≤ 0.05, ** P ≤ 0.01, *** P ≤ 0.001, and **** P ≤ 0.0001.

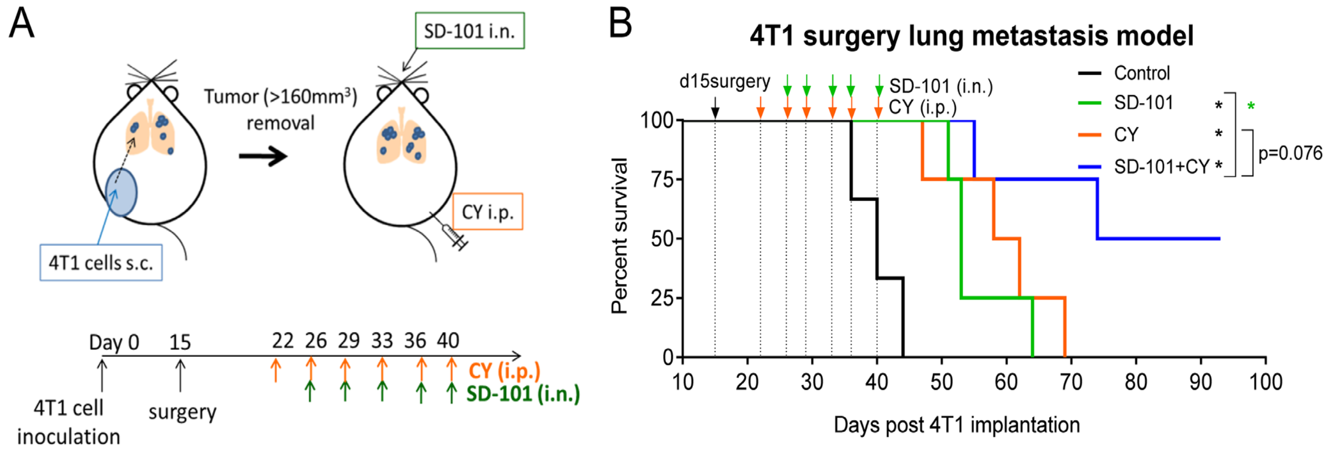

Supplementary Figure 2: Inhaled SD-101 in combination with low dose cyclophosphamide conferred increase survival in model of tumor-burdened lung. (A) Illustration of 4T1 surgery lung metastasis model. 4T1 cells (1.2×10^4) were injected s.c. and primary tumors were resected at day 15 post 4T1 implantation when they reached > 160 mm^3. Early surgical removal of the 4T1 s.c. tumor resulted in multiple metastases primarily, but not exclusively, in the lung. CY (40 mg/kg) was given i.p. and SD-101 (10 µg/50 µl saline) was given i.n., twice weekly for three weeks as indicated in (A). **(B)** Mice bearing 4T1 lung tumors were monitored for survival, n=4/group. * indicates $P \leq 0.05$, ** $P \leq 0.01$, *** $P \leq 0.001$, and **** $P \leq 0.0001$.

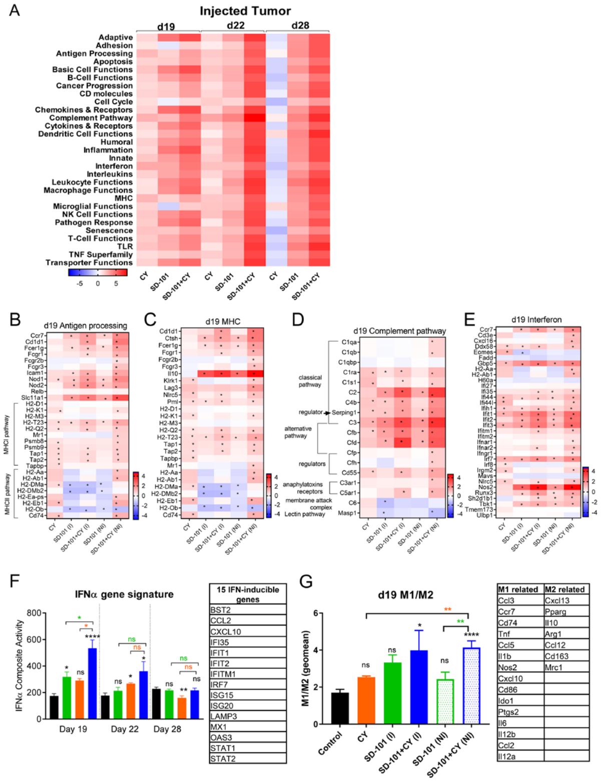

Supplementary Figure 3: NanoString analysis of the tumors in response to treatments. Experimental schema as in Figure 4A. **(A)** Heatmap plot of directed global significance scores (injected tumor). **(B–E)** Heatmaps showing relative expression levels of antigen processing, MHC, complement pathway and interferon genes at d19. * indicates a $\log_2$ fold change >0.6 and P<0.05 compared to control group at that time point. **(F)** IFNα composite score, which was generated by calculating the geometric mean of the fold activity of each of the 15 well- characterized IFN- responsive genes, of the non-injected tumors. **(G)** Ratio of the geometric mean of the tumor M1 and M2 related gene expression at day19. Data are mean ± SEM, * compared with control, * compared with SD-101, * compared with CY. * indicates P ≤ 0.05, ** P ≤ 0.01, *** P ≤ 0.001, and **** P ≤ 0.0001.

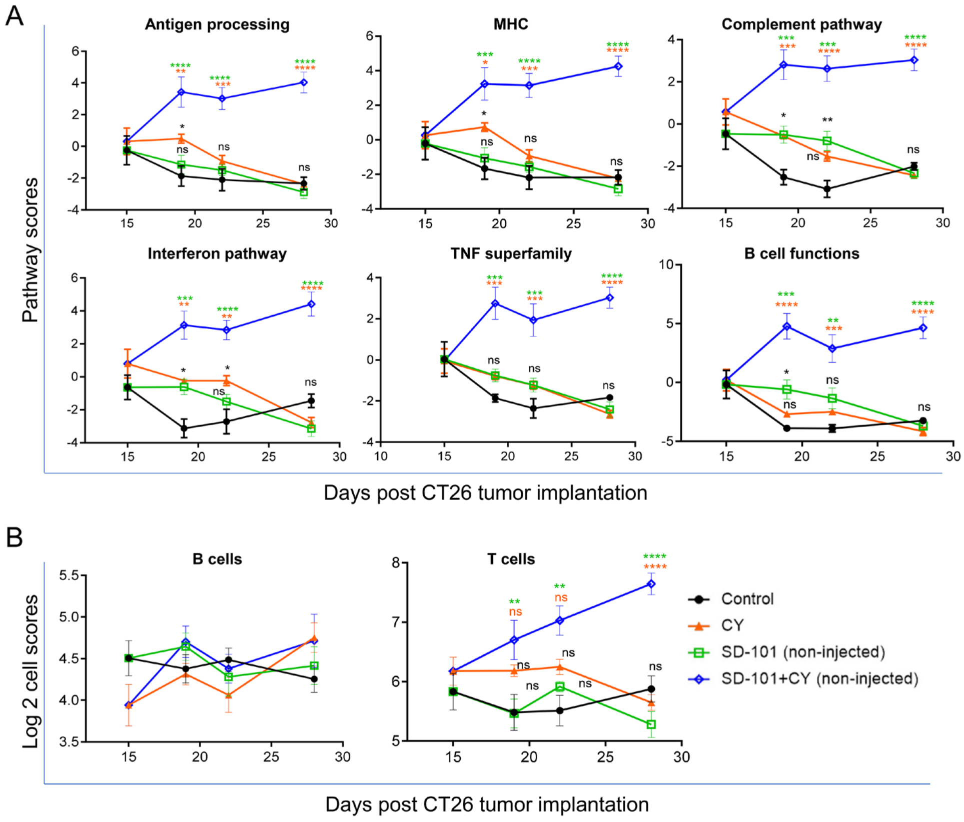

Supplementary Figure 4: Pathway and cell type scores of the non-injected tumors. Experimental schema as in Figure 4A. **(A)** Pathway scores of selected functional pathways in the non-injected tumors. **(B)** Cell type scores of B cells and T cells in the non-injected tumors. Data are mean ± SEM, * compared with control, * compared with SD-101, * compared with CY. * indicates $P \leq 0.05$, ** $P \leq 0.01$, *** $P \leq 0.001$, and **** $P \leq 0.0001$.

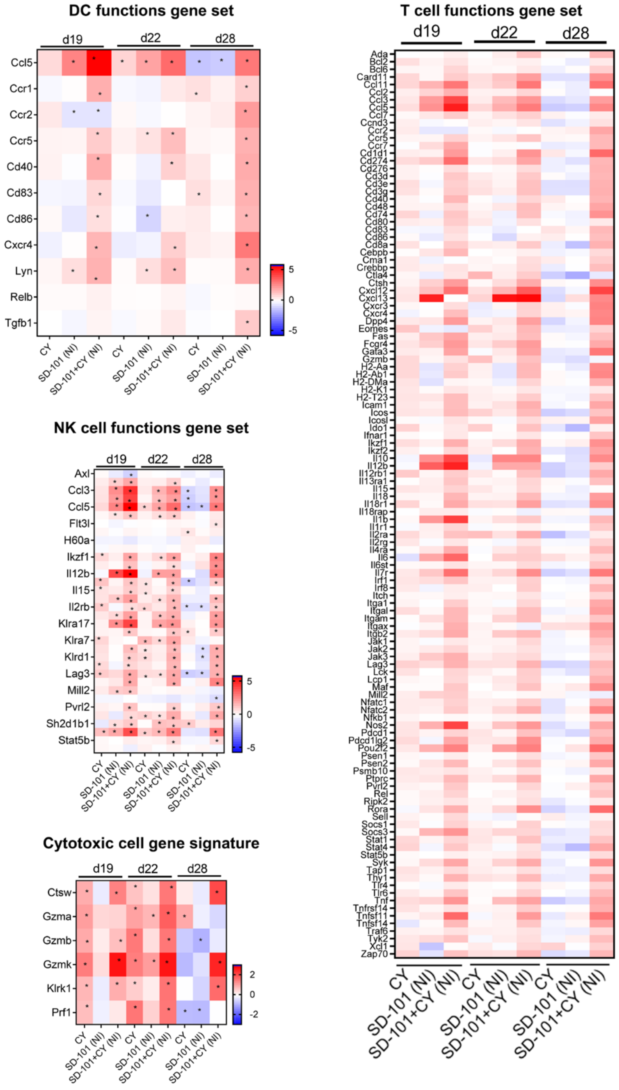

Supplementary Figure 5: Heatmaps showing relative expression levels of DC- function genes, NK- function genes, T cell- function genes and genes of cytotoxic signature. Experimental schema as in Figure 4A. * indicates a $\log_2$ fold change >0.6 and P<0.05 compared to control group at that time point.

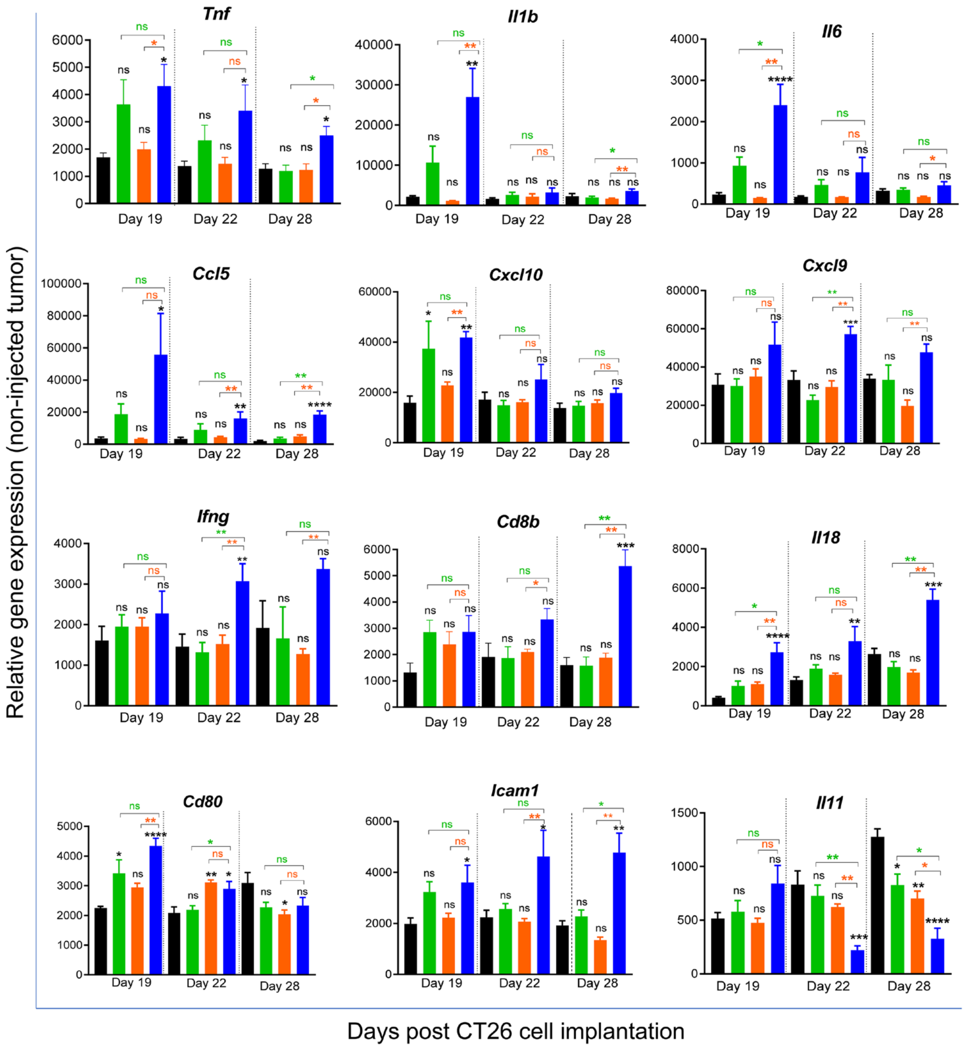

Supplementary Figure 6: Expression changes of selected genes in the non-injected tumors examined using real time qPCR. Experimental schema as in Figure 4A. Data are mean ± SEM, * compared with control at that time point, * compared with SD-101 at that time point, * compared with CY at that time point, n=6/group. * indicates P ≤ 0.05, ** P ≤ 0.01, *** P ≤ 0.001, and **** P ≤ 0.0001.

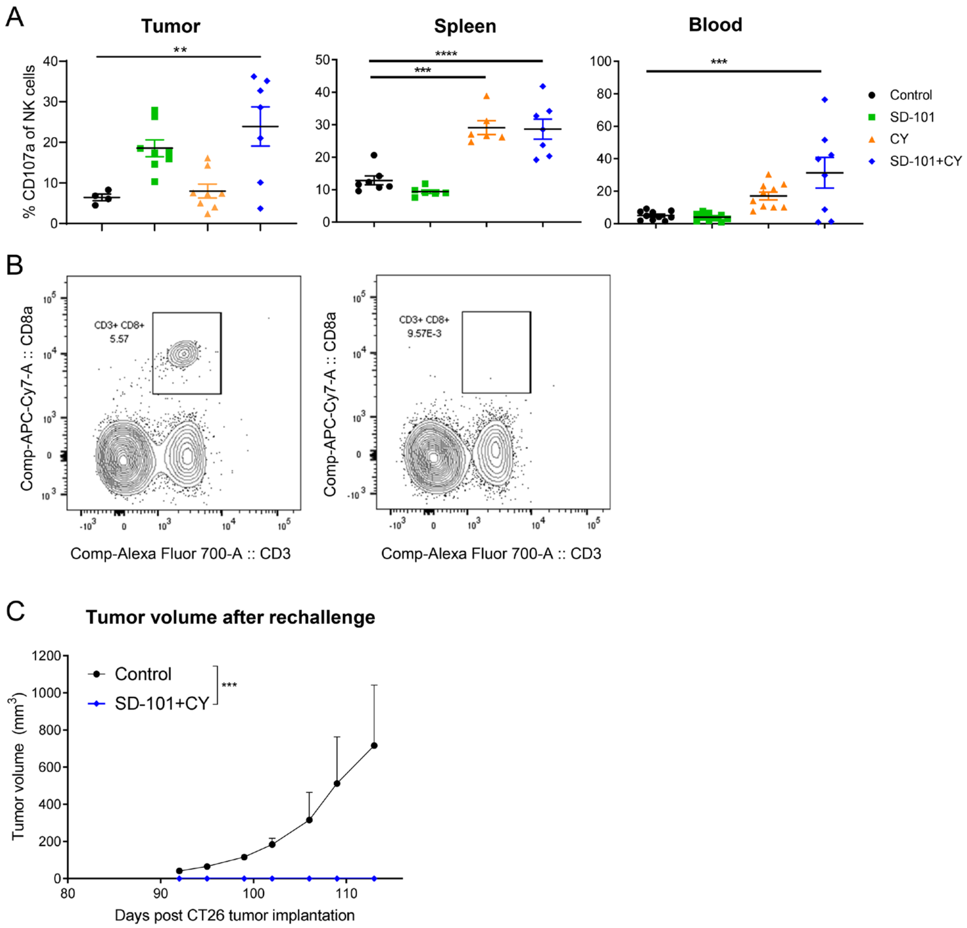

Supplementary Figure 7: (A) Combination therapy increased activity of NK and CD8+ T cells. Frequency of activated NK of total DX5+ CD3- following treatment with 3 doses CY and two doses of SD-101. **(B)** Representative plot of CD45+CD3+ CD8+ cells in the blood of mice with and without i.p. CD8+ antibody treatment. **(C)** Tumor volume following rechallenge of naïve mice (control) or mice that had cleared their tumors with the combination treatment (Figure 2D), 86 days post the first CT26 implantation. Tumor growth after rechallenge was followed for an additional 30 days. Data are mean + SEM, n=5/group. * indicates $P \leq 0.05$, ** $P \leq 0.01$, *** $P \leq 0.001$, and **** $P \leq 0.0001$.
