# Supplementary material for: Low-dose metronomic cyclophosphamide complements the actions of an intratumoral C-class CpG TLR9 agonist to potentiate innate immunity and drive potent T cell-mediated anti-tumor responses
Source: Oncotarget. 2019 Dec 31;10(68):7220–37. doi: 10.18632/oncotarget.27322 (PMC6944447; doi:10.18632/oncotarget.27322)
Supplement: Supplementary file 2 [file oncotarget-10-7220-s002.docx]

Supplemental Data.

Table S1. DE genes in combination treatment only, but not in monotherapy treatments.

| DE genes in combination treatment only, but not in mono treatments | | |
| --- | --- | --- |
| d19 | d22 | d28 |
| 194 genes | 226 genes | 322 genes |
| Abca1 | Vwf | Defb1 |
| Abl1 | Col1a1 | Col1a1 |
| Amica1 | Jam3 | Cxcl12 |
| Angpt2 | Rora | Fap |
| Apoe | Pdcd1lg2 | Col3a1 |
| App | Nfatc4 | Ccl21a |
| Arg1 | Cd1d1 | Jam3 |
| Axl | Fap | Apoe |
| Bcl6 | Ciita | Cmah |
| Birc5 | Tnfsf11 | Vwf |
| Bmi1 | Apoe | Nfatc4 |
| Bst1 | Dpp4 | Il1a |
| Btk | Mefv | Cd1d1 |
| C1qa | Gata3 | Pou2f2 |
| C1qb | Abca1 | S100a8 |
| C3ar1 | Tlr9 | Csf3r |
| C5ar1 | Cfh | Egfr |
| Card11 | Tlr1 | Ccl11 |
| Casp1 | Arg2 | Vegfc |
| Casp8 | Vegfc | Abcg1 |
| Ccl21a | Mertk | Cd36 |
| Ccl6 | Tlr8 | Gata3 |
| Ccl8 | Cxcl14 | Ciita |
| Ccl9 | Cdh1 | Cdh1 |
| Ccr1 | Ikzf2 | Tnfsf11 |
| Ccr5 | Il16 | C3 |
| Cd14 | Ltb | Cd207 |
| Cd1d1 | Arg1 | Tgfb2 |
| Cd276 | Mx2 | Il16 |
| Cd33 | Entpd1 | Irak3 |
| Cd34 | Ctss | Abca1 |
| Cd36 | Rsad2 | Cxcr3 |
| Cd37 | H2-Eb1 | Thbd |
| Cd3d | H2-Aa | Nfatc2 |
| Cd3e | Itk | H2-Ab1 |
| Cd3g | H2-Ea-ps | Cxcr4 |
| Cd40 | Csf2rb | Slamf7 |
| Cd47 | H2-Ab1 | Cd38 |
| Cd48 | Slamf6 | Smpd3 |
| Cd53 | Cd84 | Csf2rb |
| Cd68 | Il10ra | Fos |
| Cd80 | Colec12 | Itga4 |
| Cd83 | Ccrl2 | Il12b |
| Cd84 | Il3ra | Tlr1 |
| Cd86 | Il18 | Cxcl13 |
| Cd8a | Hspb2 | Ticam2 |
| Cd97 | Itgal | Colec12 |
| Cdh1 | Cxcl10 | H2-Aa |
| Cdh5 | Il1a | H2-Eb1 |
| Cfh | Eng | Maf |
| Cfp | Cd74 | Il21r |
| Chit1 | Cxcl11 | St6gal1 |
| Chuk | Cmklr1 | Arg2 |
| Cklf | Cd3e | Cd200r1 |
| Clec7a | Cybb | Ctsh |
| Cmklr1 | Psen2 | Eng |
| Colec12 | Ifi44l | H2-Ea-ps |
| Crebbp | Tgfbr2 | Il18r1 |
| Csf2rb | Maf | Chit1 |
| Ctss | Cx3cl1 | Ebi3 |
| Cxcl16 | St6gal1 | Ikzf1 |
| Cxcr4 | Ada | Epcam |
| Cxcr6 | Inpp5d | Mertk |
| Cybb | Cxcl9 | Tlr8 |
| Dpp4 | Tnfrsf1b | Emr1 |
| Dusp6 | C6 | Card11 |
| Egr1 | Tnfsf14 | Fpr2 |
| Eng | Nlrc5 | Pik3cd |
| Entpd1 | Mr1 | Cd74 |
| Ep300 | Pecam1 | Nod2 |
| Epcam | Cd53 | Cybb |
| Ewsr1 | Il1b | Cx3cl1 |
| F2rl1 | Cd68 | Syk |
| Fap | C1qb | Slc11a1 |
| Fcgr1 | Siglec1 | Cmklr1 |
| Fcgr2b | C1qa | Mef2c |
| Fcgr3 | Itga4 | Cd53 |
| Fyn | Tlr2 | Tlr9 |
| Gata3 | Cd3d | Tie1 |
| H2-Aa | Cd8a | Inpp5d |
| H2-Ab1 | Irf5 | Ltb |
| H2-Ea-ps | Tlr7 | Il10ra |
| Hcst | H2-T23 | Tnfrsf11a |
| Hif1a | Cd97 | Cyfip2 |
| Hmgb1 | Tnfrsf14 | Gzmk |
| Hspb2 | Map3k1 | Itgax |
| Icam1 | Nfatc1 | Serpinb2 |
| Icos | Lrp1 | Csf1r |
| Icosl | Klra2 | Il11ra1 |
| Ifi35 | Ifit2 | Cd84 |
| Ifitm1 | Lck | C4b |
| Ifitm2 | C5ar1 | Il3ra |
| Ifnar1 | Fcgr1 | Il18 |
| Ifnar2 | Irf8 | Igf2r |
| Ifngr1 | Mef2c | Cd7 |
| Igf2r | Hcst | Itgal |
| Ikbkb | Notch1 | Cd37 |
| Ikbkg | Tie1 | Kdr |
| Ikzf2 | Oasl1 | Cd180 |
| Il11 | Pdgfc | Klra17 |
| Il11ra1 | Irf1 | Icosl |
| Il13ra1 | Csf1r | F2rl1 |
| Il16 | Tlr6 | Tnfsf14 |
| Il17ra | Socs1 | Nlrp3 |
| Il18 | Il4ra | Ctss |
| Il1r1 | Il6ra | Selplg |
| Il1rap | Cxcr3 | Cd97 |
| Il1rl2 | Igf2r | Ccrl2 |
| Il3ra | Kdr | Ikzf2 |
| Il6ra | Ccl6 | Tlr7 |
| Il6st | Tnfrsf11b | Clec4a2 |
| Inpp5d | Ly86 | Tlr2 |
| Irf2 | Btk | Entpd1 |
| Irf5 | H2-DMa | Il13ra2 |
| Itga1 | Fyn | Cd48 |
| Itga6 | Cxcl16 | Batf |
| Itgax | Nlrp3 | Pdgfc |
| Itgb3 | Cxcr4 | Nos2 |
| Jak1 | Xcr1 | Ccr2 |
| Jak2 | Pvrl2 | Ptprc |
| Kdr | Egr3 | Notch1 |
| Klra2 | Ccl9 | Icam1 |
| Klrc1 | Nt5e | Col4a1 |
| Klrd1 | Il13ra1 | Trem1 |
| Lag3 | Ccl2 | Pik3cg |
| Lcp1 | Cd40 | Cxcl16 |
| Lif | Osm | Timd4 |
| Litaf | Tnfsf13b | Itgb2 |
| Lrp1 | Ifnar2 | Lrp1 |
| Ltb | Stat3 | Itga1 |
| Ly9 | Itga1 | Il12rb2 |
| Ly96 | Ifngr1 | Psen2 |
| Maf | Ep300 | Hck |
| Map3k1 | Jak1 | Fcgr4 |
| Map4k2 | Xcl1 | Thy1 |
| Mapk14 | Ly9 | Hsd11b1 |
| Masp1 | Snai1 | Ctsw |
| Mertk | Nrp1 | Xcr1 |
| Mfge8 | Pml | Tnfrsf13b |
| Mr1 | Trem2 | Nod1 |
| Myd88 | Ncf4 | Ly86 |
| Ncf4 | Txnip | Cd68 |
| Nefl | Crebbp | Cxcl14 |
| Nfatc1 | Tlr3 | Pdgfrb |
| Nfatc2 | Il6st | C3ar1 |
| Nfatc4 | Tnfrsf13b | Btk |
| Nt5e | Trem1 | Cdh5 |
| Pdcd1lg2 | Psmb8 | Ccl8 |
| Pdgfrb | Icosl | Fcer1g |
| Pecam1 | Cd276 | Tgfbr2 |
| Pik3cd | Fcgr3 | Lcp1 |
| Pik3cg | Hif1a | Tnfrsf1b |
| Plaur | Stat5b | Tnfrsf14 |
| Prdm1 | Prkcd | H2-DMa |
| Prkcd | Fcer1a | Cfp |
| Ptgs2 | Timd4 | Il6ra |
| Pvrl2 | Muc1 | Sh2d1a |
| Rel | Cma1 | Egr3 |
| Ripk2 | Casp1 | Cd3e |
| Rora | Tap1 | Irf5 |
| Rrad | Tyk2 | Bst1 |
| Sell | Psen1 | Cfb |
| Sh2b2 | Kit | Ikbke |
| Sh2d1a | Tnfsf12 | Cd4 |
| Smpd3 | Il11ra1 | Mr1 |
| Snai1 | Tfeb | Cd276 |
| Socs1 | Ly96 | Pecam1 |
| Stat3 | Atm | Ifngr1 |
| Stat4 | Ripk2 | Nlrc5 |
| Tek | Col4a1 | Lyn |
| Tfeb | Pdgfrb | Cd40 |
| Tgfbr2 | Myd88 | Nt5e |
| Thy1 | Psmb9 | Irf8 |
| Ticam2 | Jak2 | Prkcd |
| Tlr1 | Cdh5 | Map3k1 |
| Tlr2 | Irak4 | Nfatc1 |
| Tlr6 | Irf2 | Nrp1 |
| Tlr7 | Psmb10 | Cd3d |
| Tnfrsf11a | Traf6 | Ada |
| Tnfrsf12a | Tnfrsf1a | Fcgr1 |
| Tnfrsf1a | Rel | Il1b |
| Tnfrsf1b | Tpsab1 | Il4ra |
| Tnfsf11 | Ikbkg | Ncf4 |
| Tnfsf14 | Tlr4 | Cd83 |
| Tollip | Fadd | Tnfsf13b |
| Traf6 | Litaf | Alcam |
| Trem1 | Itch | Ccr5 |
| Twist1 | Ccnd3 | Ccr7 |
| Txnip | Raet1c | Klrk1 |
| Ubc | Il2rg | C5ar1 |
| Vegfc | Lamp2 | C1qb |
| Vwf | App | Tlr6 |
| Xbp1 | Cyld | Amica1 |
| Zap70 | Ifi35 | Ifnar2 |
|  | Traf3 | Tyk2 |
|  | Ifnar1 | Itgae |
|  | Tmem173 | Crebbp |
|  | Ikbkb | Fyn |
|  | H2-K1 | Arg1 |
|  | Tap2 | Cd55 |
|  | Il17ra | Cd86 |
|  | Vcam1 | Fas |
|  | Creb5 | Cxcl9 |
|  | Il1rl2 | Il17ra |
|  | Blnk | Il13ra1 |
|  | Gtf3c1 | Jak1 |
|  | Tgfbr1 | Snai1 |
|  | Casp8 | Il15ra |
|  | Mapk14 | Itgb3 |
|  | Msr1 | Rel |
|  | Bcl6 | Ppbp |
|  | Tfe3 | Hif1a |
|  | Igf1r | Fcgr2b |
|  | Alcam | Icam2 |
|  | Map2k4 | Il6st |
|  | Cd164 | Ly9 |
|  | Lamp1 | Fadd |
|  | Pvr | Ifnar1 |
|  | Ptgs2 | Stat1 |
|  | Il7 | C1qa |
|  | Itga6 | H2-T23 |
|  | Cd9 | Ep300 |
|  | Sigirr | Sbno2 |
|  | Lrrn3 | Itgam |
|  | Tnfrsf12a | Pin1 |
|  | Nefl | Psen1 |
|  |  | Lcn2 |
|  |  | Tgfbr1 |
|  |  | Cd69 |
|  |  | Il1rap |
|  |  | Stat3 |
|  |  | Il1r1 |
|  |  | Tfeb |
|  |  | Siglec1 |
|  |  | Raet1c |
|  |  | Slc7a11 |
|  |  | Tgfb3 |
|  |  | Litaf |
|  |  | Ccl6 |
|  |  | Ikbkb |
|  |  | App |
|  |  | Cma1 |
|  |  | Gpr183 |
|  |  | Pvrl2 |
|  |  | Ticam1 |
|  |  | Jak2 |
|  |  | Cd14 |
|  |  | Tgfb1 |
|  |  | Ifit2 |
|  |  | Bcl6 |
|  |  | Myd88 |
|  |  | Map4k2 |
|  |  | Mapk14 |
|  |  | Irf2 |
|  |  | Ly96 |
|  |  | Il2rg |
|  |  | Eomes |
|  |  | Tlr4 |
|  |  | Msr1 |
|  |  | Cxcl11 |
|  |  | Abl1 |
|  |  | Irak4 |
|  |  | Tnfrsf1a |
|  |  | Lamp2 |
|  |  | Il1rl2 |
|  |  | Gtf3c1 |
|  |  | Itch |
|  |  | Plau |
|  |  | Irf1 |
|  |  | Map3k5 |
|  |  | Tnfsf12 |
|  |  | Runx1 |
|  |  | Traf6 |
|  |  | Socs1 |
|  |  | Cebpb |
|  |  | Txk |
|  |  | Xbp1 |
|  |  | Cxcl10 |
|  |  | Map2k4 |
|  |  | Jak3 |
|  |  | Nfatc3 |
|  |  | Atm |
|  |  | Casp8 |
|  |  | Stat5b |
|  |  | Cd164 |
|  |  | Nfkb1 |
|  |  | Tbk1 |
|  |  | Ccl9 |
|  |  | Tap1 |
|  |  | Txnip |
|  |  | Mapkapk2 |
|  |  | Tirap |
|  |  | Atg5 |
|  |  | Tfe3 |
|  |  | Dock9 |
|  |  | Creb1 |
|  |  | Lamp1 |
|  |  | Sh2b2 |
|  |  | Cd200 |
|  |  | Ets1 |
|  |  | Psmb8 |
|  |  | Stat6 |
|  |  | Ifitm2 |
|  |  | Psmb10 |
|  |  | H2-K1 |
|  |  | Tollip |
|  |  | Map2k2 |
|  |  | Atg10 |
|  |  | Mif |
|  |  | Plaur |
|  |  | Il34 |
|  |  | Pvr |
|  |  | Cd9 |
|  |  | Cd34 |
|  |  | Sigirr |
|  |  | Il7 |
|  |  | Abcb1a |
|  |  | Tnfrsf12a |
|  |  | Il11 |
|  |  | Lrrn3 |
|  |  | Nefl |
|  |  | Sele |
